# Solution structure of the autophagy-related protein LC3C reveals a polyproline II motif on a mobile tether with phosphorylation site

**DOI:** 10.1038/s41598-019-48155-8

**Published:** 2019-10-02

**Authors:** Carsten Krichel, Christina Möckel, Oliver Schillinger, Pitter F. Huesgen, Heinrich Sticht, Birgit Strodel, Oliver H. Weiergräber, Dieter Willbold, Philipp Neudecker

**Affiliations:** 1 ICS-6 (Strukturbiochemie) and JuStruct, Forschungszentrum Jülich, 52425 Jülich, Germany; 2 Institut für Physikalische Biologie and BMFZ, Heinrich-Heine-Universität Düsseldorf, 40225 Düsseldorf, Germany; 3 Institut für Theoretische Chemie und Computerchemie, Heinrich-Heine-Universität Düsseldorf, 40225 Düsseldorf, Germany; 4 ZEA-3 (Analytik), Forschungszentrum Jülich, 52425 Jülich, Germany; 5 Institut für Biochemie, Friedrich-Alexander-Universität Erlangen-Nürnberg, 91054 Erlangen, Germany

**Keywords:** Solution-state NMR, Molecular modelling

## Abstract

(Macro-)autophagy is a compartmental degradation pathway conserved from yeast to mammals. The yeast protein Atg8 mediates membrane tethering/hemifusion and cargo recruitment and is essential for autophagy. The human MAP1LC3/GABARAP family proteins show high sequence identity with Atg8, but MAP1LC3C is distinguished by a conspicuous amino-terminal extension with unknown functional significance. We have determined the high-resolution three-dimensional structure and measured the backbone dynamics of MAP1LC3C by NMR spectroscopy. From Ser18 to Ala120, MAP1LC3C forms an α-helix followed by the ubiquitin-like tertiary fold with two hydrophobic binding pockets used by MAP1LC3/GABARAP proteins to recognize targets presenting LC3-interacting regions (LIRs). Unlike other MAP1LC3/GABARAP proteins, the amino-terminal region of MAP1LC3C does not form a stable helix α_1_ but a “sticky arm” consisting of a polyproline II motif on a flexible linker. Ser18 at the interface between this linker and the structural core can be phosphorylated *in vitro* by protein kinase A, which causes additional conformational heterogeneity as monitored by NMR spectroscopy and molecular dynamics simulations, including changes in the LIR-binding interface. Based on these results we propose that the amino-terminal polyproline II motif mediates specific interactions with the microtubule cytoskeleton and that Ser18 phosphorylation modulates the interplay of MAP1LC3C with its various target proteins.

## Introduction

**Figure 1:**
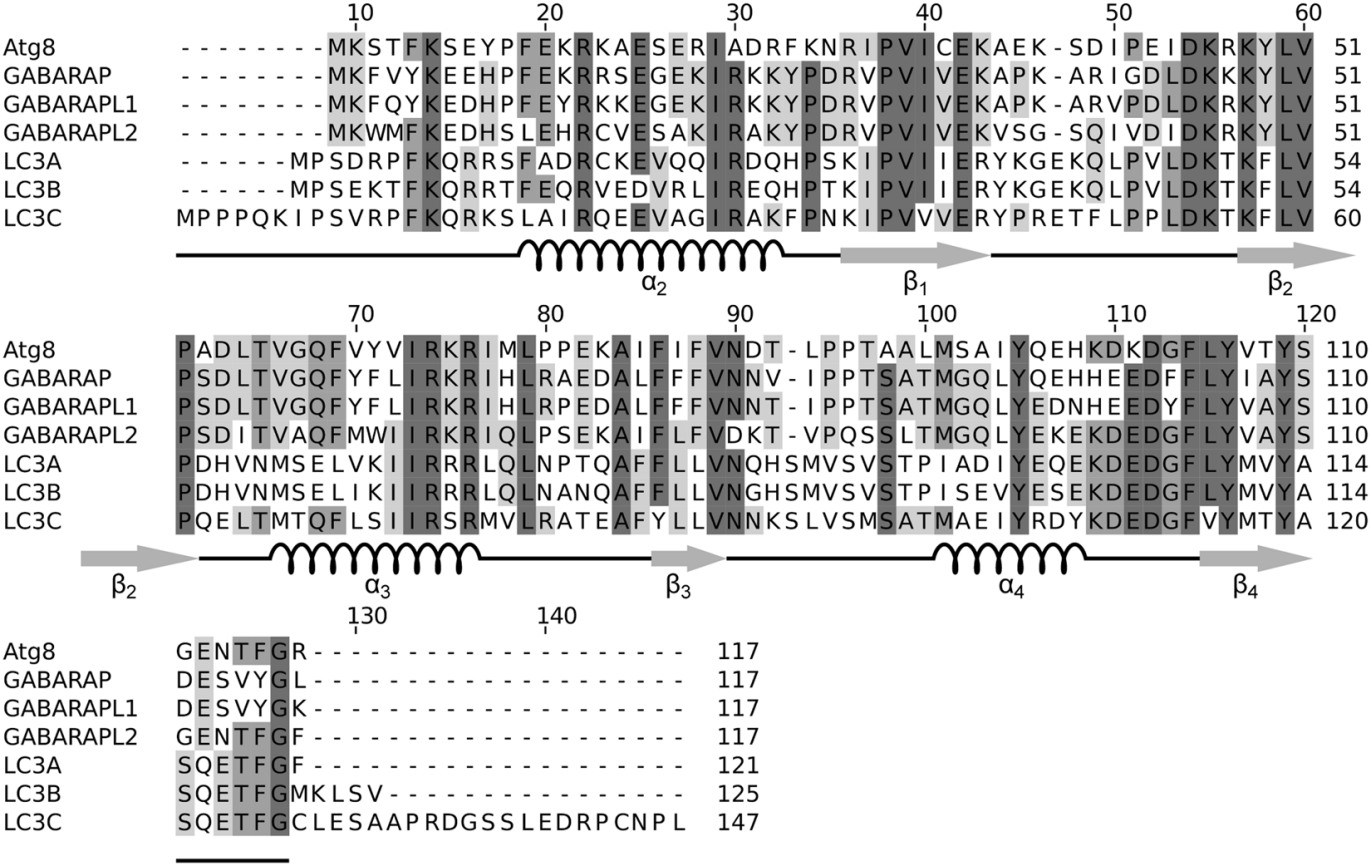
Sequence alignment of *S*. *cerevisiae* Atg8 (Uniprot P38182) and the canonical human homologues GABARAP (O95166), GABARAPL1/GEC1 (Q9H0R8), GABARAPL2/GATE-16 (P60520), and LC3A (Q9H492), LC3B (Q9GZQ8), and LC3C (Q9BXW4). Highly conserved residues are highlighted in shades of gray; darker shading indicates higher conservation. All members of the Atg8 family can be cleaved by the cysteine protease family ATG4 after the conserved Gly126, which can subsequently be conjugated to a phospholipid moiety for membrane anchoring. The alignment was performed using Jalview version 2^98^. The regular secondary structure elements of LC3C are indicated below the alignment.

Macroautophagy - hereafter termed autophagy - is an intracellular lysosomal degradation pathway conserved in eukaryotes^1^. Upon initiation of autophagy a newly emerging double membrane structure, the phagophore, engulfs cytoplasmatic targets and closes to form a double membrane vesicle, the autophagosome, which then fuses with lysosomal organelles for cargo degradation. The protein Atg8 (autophagy-related 8) is essential for autophagosome genesis in the yeast *Saccharomyces cerevisiae*^2^. The carboxy-terminal residue of Atg8, Arg117 (Fig. 1), is removed by the *S*. *cerevisiae* cysteine protease Atg4 to expose a conserved glycine, Gly116, at the C-terminus. Subsequently, this exposed glycine can enzymatically be conjugated covalently to the membrane lipid phosphatidylethanolamine and thereby tethered to the growing phagophore^3^.

In humans, the Atg8 gene has diversified into at least eight orthologs, which can be divided into two subfamilies: the GABARAP (γ-aminobutyric acid type A receptor associated protein)-like and MAP1LC3 (microtubule associated proteins 1 light chain 3)-like proteins^4,5^. The functional role of this diversification is still poorly understood. Like Atg8, these proteins are initially expressed as longer precursor proteins and then cleaved by the *H*. *sapiens* ATG4 family of cysteine proteases to expose the conserved glycine (Gly126 in the case of MAP1LC3C; Fig. 1) at the C-terminus. The high-resolution three-dimensional structure of several of these proteins has been determined experimentally by X-ray crystallography and/or NMR spectroscopy (reviewed by Weiergräber *et al*.^5^). In particular, solution structures have been reported for yeast Atg8^6–8^, for the human GABARAP-like proteins GABARAP^9^ and GABARAPL1^10^, and for the MAP1LC3-like proteins MAP1LC3A^11^, MAP1LC3A in complex with SQSTM1^12^, and MAP1LC3B in complex with optineurin^13^. Recently, crystal structures for MAP1LC3C in the free form^14^ (PDB 3WAM) as well as in complex with Atg13^14^ (PDB 3WAP) and in complex with NDP52^15^ (PDB 3VVW) were published. All these Atg8 homologs share a ubiquitin-like core structural motif consisting of a four-stranded β-sheet flanked by two α-helices, which is extended by an amino-terminal α-helical domain. The human MAP1LC3 (or simply LC3) proteins differ most significantly in the amino-terminal region (NTR) preceding this α-helical domain, which is considerably longer in LC3C as compared to LC3A and LC3B (Fig. 1). While for the latter two proteins a role of the NTR in membrane tethering^2,16^ or in recognition of mitochondrial phospholipids during mitophagy has been reported^17^, the functional implications of the longer NTR found in LC3C are still poorly understood.

Autophagy research has historically focused on unspecific (bulk) degradation of cytosolic targets. Recently, however, mammalian autophagy receptor proteins have been described that mediate selective autophagic pathways^18,19^. Recruitment of selective autophagic degradation targets to LC3-like proteins is mediated by the target’s conserved LC3-interacting region (LIR) of four amino acids with a canonical sequence motif (W/Y/F-x-x-L/I/V)^12,18,20^. In contrast to other LC3 proteins, LIR binding to LC3C in selective autophagy can also be mediated by a non-canonical LIR motif (CLIR)^15^. Upon binding to LC3 the two large hydrophobic side chains flanking the LIR of the target insert into two hydrophobic pockets on the surface of LC3 and the backbone of the LIR forms an additional, parallel β-strand to extend the core LC3 β-sheet separating the two hydrophobic pockets^12^.

Autophagy is regulated by a variety of mechanisms, including the modulation of LC3 protein-protein interactions via post-translational modifications. On the one hand, the affinity of binding to LC3 can be enhanced by modification of the target LIR^19,21^. On the other hand, post-translational modifications of the LC3 proteins themselves have been described. Intriguingly, the post-translational modification sites are predominantly localized within the NTR of LC3. Cherra *et al*.^22^ reported the down-regulation of autophagy upon phosphorylation of LC3A at Ser12 by protein kinase A (PKA), while Jiang *et al*.^23^ studied the influence of phosphorylation of LC3B at Thr6 and Thr26 by protein kinase C (PKC) on autophagosome formation. Both studies concluded that an increased cellular protein kinase activity could attenuate autophagy. Structural studies, however, were beyond the scope of these works. LC3C shares the PKA phosphorylation site mentioned above (Ser18 in this case) with LC3A and LC3B^22^, and, unlike other LC3-proteins, contains a second PKC phosphorylation site at Ser9 as predicted by computational algorithms^24^. Furthermore, Huang *et al*.^25^ reported the influence of de-acetylation of a lysine residue in nuclear-localised LC3, suggesting further modulating options for LC3 activity in its interplay with the autophagic machinery. Other diverse post-translational modifications of autophagy-related proteins have been reviewed recently^26^.

In order to provide a structural basis for understanding the functional role of the NTR of LC3C on the atomic level we have determined the three-dimensional structure of human MAP1LC3C directly in solution and investigated the backbone dynamics and the influence of *in vitro* phosphorylation by PKA using NMR spectroscopy.

## Results and Discussion

### Description of the structure in solution

As reported previously^27^, inspection of the NMR spectra and analysis of the ^15^N relaxation and relaxation dispersion experiments (see below) reveal that LC3C exhibits extensive conformational heterogeneity in several regions of the protein, which results in significant conformational averaging and/or exchange line broadening of the NMR spectra (Supplementary Fig. S3). Accordingly, manual inspection of the experimental restraints was necessary to exclude artificially restrictive restraints derived from conformationally averaged spectral parameters such as NOEs (Supplementary Fig. S4) or ^3^J_HNH__α_ couplings. An ensemble of ten solution structures was calculated from 1162 NOE distance restraints, 214 dihedral angle restraints, and 102 hydrogen bond distance restraints. Of note, all three α-helices feature a slowly exchanging backbone amide hydrogen donor involved in N-capping hydrogen bonds to side chain oxygen atoms (α_2_: Ile21 HN – Ser18 Oγ; α_3_: Gln68 HN – Thr65 Oγ1; α_4_: Glu103 HN – Thr100 Oγ1). Experimental restraints and statistics of the structural ensemble are summarized in Table 1.

**Table 1:** Statistics of the NMR ensemble (n = 10).

Statistics of the NMR ensemble	
Conformational restraints	
NOE distance restraints	
Total	1162
Intraresidual (i = j)	202
Sequential (|i − j| = 1)	387
Medium range (1 < |i − j| < 5)	266
Long range (|i − j| ≥ 5)	307
Ambiguous	57
Hydrogen bond distance restraints	102
Dihedral angle restraints	214
Residual restraint violations	
Average number of distance restraint violations per structure	
>0.1 Å	7.8
>0.3 Å	0.2 (max. 0.34 Å)
Average number of dihedral angle restraint violations	
>1°	10.2
>3°	0.4 (max. 3.77°)
Atomic RMSDs from the average structure^a^ **[Å]**	
Backbone atoms	0.72 ± 0.10
Heavy atoms	1.30 ± 0.12
MolProbity Ramachandran statistics [%]	
Favored regions	91.6
Allowed regions	99.2
Model content	
Total no. of residues	126
Ordered residue range	18–120
BMRB accession number	26603
PDB ID code	2NCN

^a^Ordered residue range (18–120).

**Figure 2:**
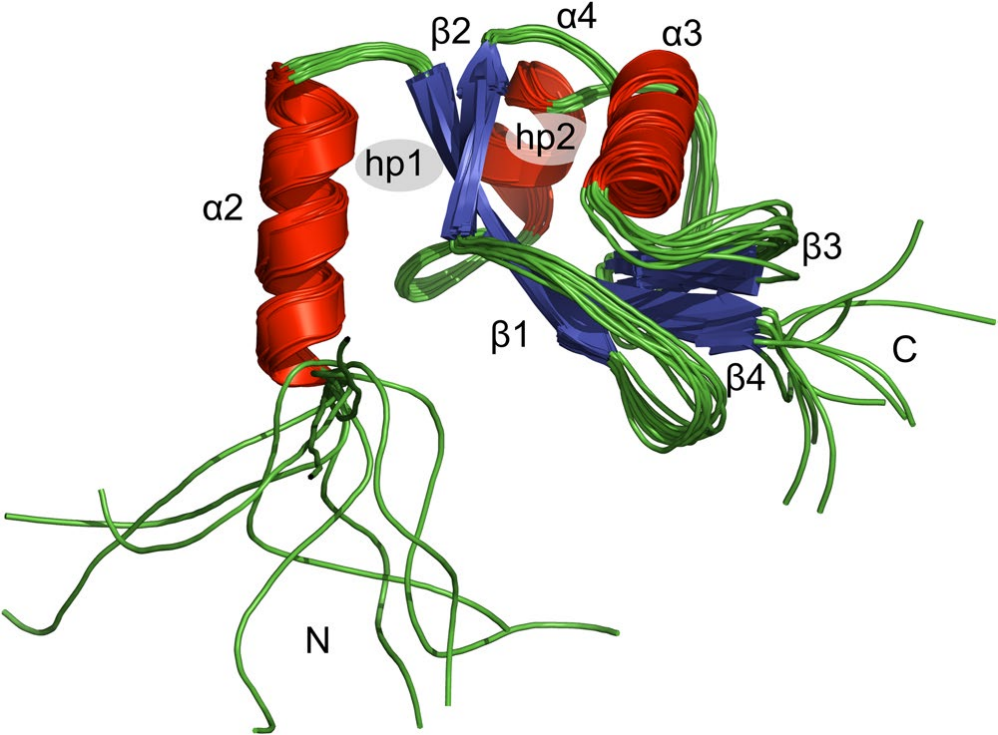
Solution structure of LC3C shown as a superposition of the backbone traces of the 10 accepted structures with a schematic representation of the secondary structure elements as identified with DSSP^99^. The tertiary structure of LC3C consists of three α-helices (α_2_: Leu19 to Lys32, α_3_: Met66 to Arg76, α_4_: Met101 to Tyr108; red) and a central β-sheet of four β-strands (β_1_: Lys36 to Arg43, β_2_: Lys57 to Pro61, β_3_: Tyr86 to Val89, β_4_: Val115 to Ala120; blue). In the ubiquitin-like core of the protein the 10 accepted structures are in excellent agreement, whereas the amino (N) and carboxy (C) termini as well as some of the loops are highly flexible in solution. The location of the two hydrophobic pockets is indicated by hp1 and hp2. The overlay was performed using PyMOL (The PyMOL Molecular Graphics System, Version 1.7.2.1, Schrödinger, LLC).

The overall structure of LC3C features a globular, ubiquitin-like fold consisting of four β-strands, three α-helices, and polymorphic terminal regions (see Fig. 2). The core of LC3C is formed by a twisted, mixed parallel/antiparallel β-sheet consisting of two inner β-strands (β_1_, β_4_) pairing in a parallel orientation, each paired by an antiparallel β-strand (β_2_ and β_3_, respectively) on the outside. The three α-helices α_2_, α_3_, and α_4_ (this numbering has been chosen for consistency with other members of the Atg8 family that have an additional α-helix at the N-terminus) are flanking the inner β-sheet on both faces, with helix α_3_ in an orientation that is approximately perpendicular to that of α_2_ and α_4_. It is this arrangement of the regular secondary structure elements that forms the two characteristic hydrophobic surface pockets, hp1 between α_2_ and β_2_ on one face of the β-sheet and hp2 between α_3_ and β_2_ on the other face (Fig. 2), which allow Atg8 family proteins to bind canonical or non-canonical LC3-interacting regions (LIRs) via β-strand pairing of the LIR backbone to the edge strand β_2_ while accommodating the two bulky hydrophobic side-chains of the LIR in hp1 and hp2^12^. The side-chain of Lys55, which is located just N-terminal of strand β_2_ and has been reported to restrict access to hp2 in order to contribute to LIR recognition^14^, is partially solvent-exposed in solution and sufficiently flexible across the ensemble of structures to allow the formation of a complex with a specifically recognized ligand (not shown).

The tertiary structure of LC3C is stabilized not just by hydrogen bonds but also by a variety of hydrophobic interactions involving aliphatic and aromatic side-chains. Tertiary contacts with aromatic side-chains give rise to several readily identifiable ^1^H chemical shift outliers due to ring current effects. Most notably, the backbone amide proton of Arg46 displays a remarkable upfield shift (5.50 ppm), which is caused by the vicinity to the aromatic side-chain of Tyr44 and has been reported previously^27^. Unfortunately, this chemical shift is too close to the residual ^1^H_2_O signal (4.82 ppm at 20.0 °C) not to be affected by band-selective water flip-back pulses^28^ and the amide resonance of Arg46 is therefore visible only with artificially reduced intensity in amide-detected NMR experiments (Supplementary Fig. S3). Additional upfield shifts of ^1^H chemical shifts were also identified for one of the two methyl groups of Val40 (−0.36 ppm; Supplementary Fig. S4), which is positioned in between the aromatic side chains of Phe114 and Tyr116, for the ^1^Hδ1 methyl group of Ile73 (−0.15 ppm), which is oriented towards Phe69, as well as for one of the two methyl groups of Val26 (0.18 ppm; Supplementary Fig. S4), which faces the aromatic ring of Phe114, and for one of the two protons of the ^1^Hδ methylene group of Lys109 (0.68 ppm), which is close to Tyr105. Strong downfield shifts of ^1^H chemical shifts were found for one of the two protons of the ^1^Hβ methylene group of Asp110 (3.79 ppm), which is in the vicinity of the aromatic side-chain of Tyr116, for Thr118 ^1^Hα (6.28 ppm), which is adjacent to the aromatic side-chain of Tyr119, and for Tyr119 ^1^Hα (6.15 ppm) itself.

### Conformational dynamics

**Figure 3:**
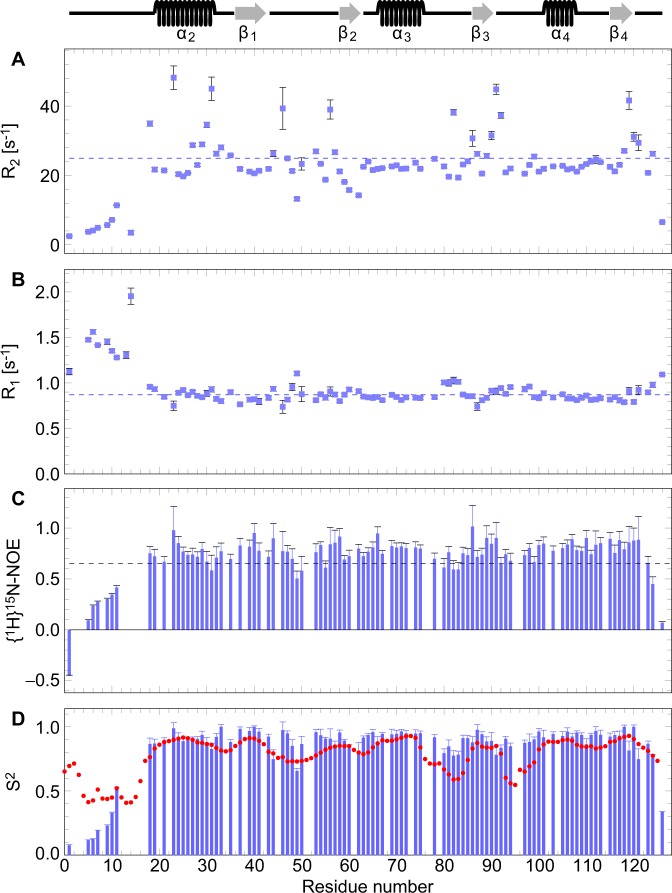
^15^N transverse (**A**) and longitudinal (**B**) relaxation rates and {^1^H}^15^N heteronuclear NOE values (**C**) measured at 800 MHz and 20.0 °C, and generalized order parameters S^2^ of the sub-nanosecond backbone amide motion (**D**). Dashed horizontal lines in (**A**,**B**) represent the average values over the ordered region (residues 18–120). {^1^H}^15^N values below 0.65 (dashed horizontal line in (**C**)) indicate increased internal mobility^29^. Experimental order parameters S^2^ obtained from ^15^N relaxation analysis (blue) are compared with order parameters predicted from the Random Coil Index (RCI)^33^, S_RCI_^2^ (red circles), calculated with the default parameters as implemented in TALOS-N^80,81^. The regular secondary structure elements are indicated above the graph.

While the structural core from Ser18 to Ala120 is well defined in solution with an average atomic RMSD from the average structure of 0.72 Å for the backbone and 1.30 Å for all heavy atoms (Tab. 1), several regions of the protein are constrained by only very few long-range NOE restraints and therefore show markedly increased atomic RMSDs (Supplementary Fig. S5), namely the N-terminus (Met1 to Lys17), the C-terminus (Ser121 to Gly126), and – to a lesser extent – the loops between strands β_1_ and β_2_ (Tyr44 to Thr56), between helix α_3_ and strand β_3_ (Met77 to Phe85), and between strand β_3_ and helix α_4_ (Asn90 to Ala99). Inspection of the ^15^N relaxation data (Fig. 3) reveals that these regions are indeed highly flexible as indicated by several {^1^H}^15^N NOE values below 0.65^29^, ^15^N transverse relaxation rates R_2_ significantly slower than the average over residues Ser18 to Ala120 of 25.0/s ± 6.7/s, and ^15^N longitudinal relaxation rates R_1_ significantly faster than the average over residues Ser18 to Ala120 of 0.87/s ± 0.07/s (Fig. 3). Accordingly, “model-free” analysis^30^ of the ^15^N relaxation data reveals large-amplitude backbone motions on the sub-nanosecond time-scale that are reflected in low generalized order parameters S^2^ at both termini and in some loop regions (Fig. 3D). By contrast, the regular secondary structure elements are quite rigid on the sub-nanosecond time-scale, with S^2^ values consistently exceeding 80%. The ratios between ^15^N transverse and longitudinal relaxation rates of the rigid structural core of LC3C at 800 MHz are best fit by an oblate axially symmetric rotational diffusion tensor with eigenvalues of 1.39 × 10^7^/s and 1.30 × 10^7^/s at 20.0 °C, corresponding to overall rotational autocorrelation times^31^ of τ_A_ = 12.0 ns, τ_B_ = 12.1 ns, and τ_C_ = 12.6 ns. In the approximation of fully isotropic rotational diffusion this reduces to a single autocorrelation time of τ_iso_ = 12.3 ns. Although prediction of the hydrodynamics of LC3C on the basis of its tertiary structure is not straightforward because of the flexibility of the long terminal regions, these autocorrelation times are slightly longer than but still consistent with the hydrodynamic properties expected for a predominantly monomeric protein the size of LC3C in the relatively viscous buffer used here. GABARAP, which is slightly smaller than LC3C (Fig. 1) and whose NTR is less mobile, shows an overall rotational autocorrelation time at 20 °C of 9.2 ns ± 0.4 ns at low concentration and about 11 ns under conditions typically used for NMR spectroscopy^32^.

**Figure 4:**
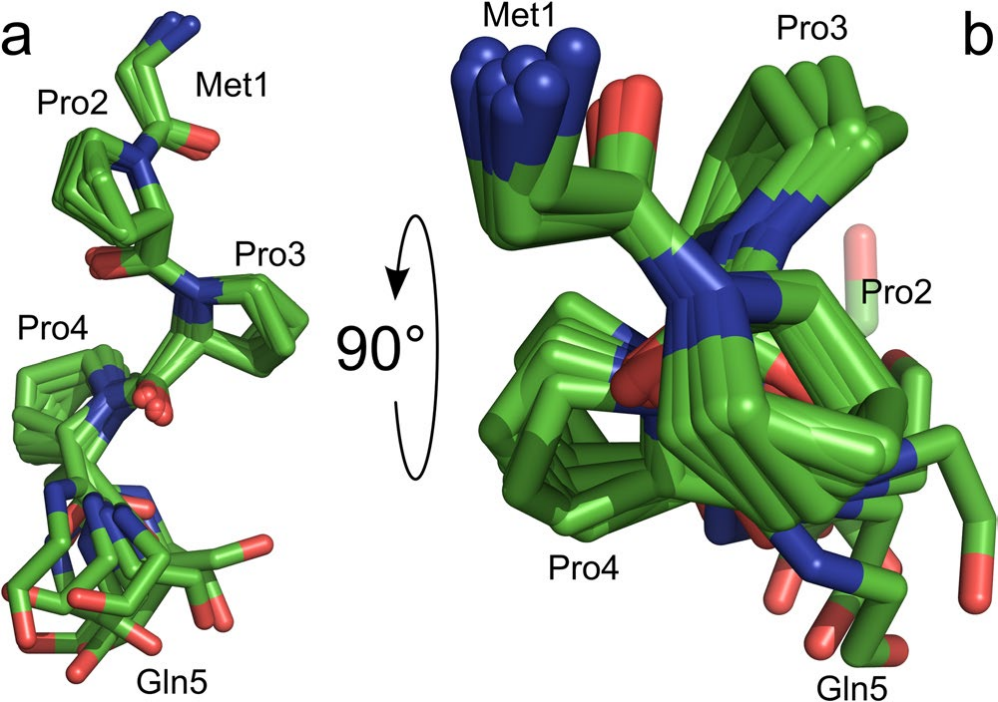
The amino-terminal polyproline II motif of LC3C shown as a superposition of the 10 accepted structures. Only the backbone atoms of the two adjacent residues, Met1 and Gln5, are shown. Carbon, nitrogen, and oxygen atoms are color-coded green, blue, and red, respectively. (**A**) Side view, (**B**) view from Met1 along the helix axis showing the typical cloverleaf-like arrangement of the proline rings due to the three-fold symmetry of the left-handed PPII helix. Pairwise alignment of backbone atoms from Pro2 to Pro4 was performed using PyMOL (The PyMOL Molecular Graphics System, Version 1.7.2.1, Schrödinger, LLC).

In addition to the generalized order parameters S^2^ from ^15^N relaxation, which are a measure of the motional restriction of the amide bond vector orientation with respect to the molecular frame on the sub-nanosecond time-scale on a scale of 0 (unrestricted motion) to 1 (rigid), we also calculated the Random Coil Index (RCI)^33^ order parameters, S_RCI_^2^, which are a measure of how different the backbone chemical shifts are from those of a disordered random coil on a scale of 0 (typical for a random coil) to 1 (typical for a well-ordered backbone conformation). In general, the generalized order parameters from ^15^N relaxation, S^2^, are in good agreement with the RCI order parameters, S_RCI_^2^, with one very conspicuous exception (Fig. 3D): Although the orientation of the NTR relative to the structural core of the molecule is largely disordered (S^2^ < 50%), the local backbone conformation of the first four residues is not consistent with a random coil (S_RCI_^2^ > 60%). Instead, a local backbone overlay of these residues reveals that the three consecutive proline residues Pro2 to Pro4 adopt a canonical polyproline II (PPII) helical geometry (Fig. 4) with its typical backbone torsion angles Φ ≈ −75° and Ψ ≈ +120° (Supplementary Fig. S6). From a structural point of view, the conformation of the unique NTR of LC3C (Fig. 1) in solution can therefore be described as an amino-terminal PPII motif attached to the ubiquitin-like core of the protein via a highly flexible 13-residue tether.

**Figure 5:**
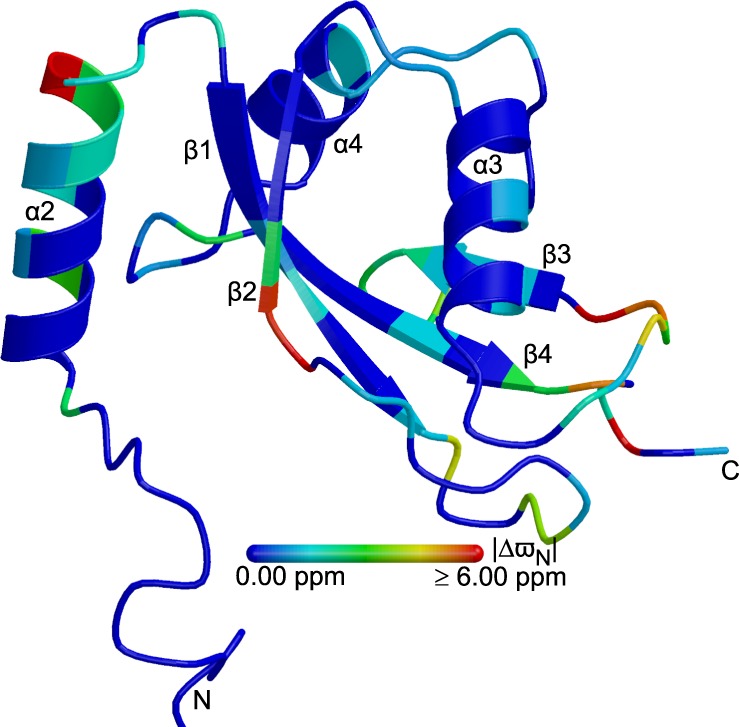
Schematic representation of the secondary structure of the lowest-energy solution structure of LC3C (similar view as in Fig. 2) colored according to the backbone amide ^15^N chemical shift change between the major (population 98.37% ± 0.06%) and the minor (population 1.63% ± 0.06%) state, |Δϖ_N_|, from 0.00 ppm (blue) to 6.00 ppm (red), or blue if Δϖ_N_ could not be determined due to missing resonance assignments, resonance overlap, or if the exchange contributions to transverse relaxation were of insufficient magnitude. Values of |Δϖ_N_| > 1.5 ppm (green to red) are localized to specific regions, indicating that the rest of the tertiary structure is not affected by the conformational exchange process. The figure was drawn with MolScript 2.1.2^100^ and rendered with Raster3D 3.0^101^.

Whereas R_1_ is solely sensitive to fast dynamics in the ns to ps regime, R_2_ is also influenced by slower conformational exchange processes on the μs to ms time-scale^34^. Inspection of the ^15^N R_2_ rates indeed shows many residues with ^15^N transverse relaxation rates that are highly elevated by conformational exchange contributions (Fig. 3A). More detailed analysis using the “model-free” formalism^30^ reveals that a large number of backbone amide groups covering virtually the entire structural core of the protein are affected by one or more conformational exchange processes on the μs to ms time-scale (data not shown), most prominently helix α_2_ (especially its C-terminal side), the loop connecting strands β_1_ and β_2_, the loops before and after strand β_3_, and the C-terminal region (Fig. 3A). These “hotspots” are corroborated by ^15^N CPMG relaxation dispersion experiments (Fig. 5). 57 backbone amide groups exhibit measurable and significant ^15^N relaxation dispersions, which can be fit by a simultaneous two-state model with an exchange rate of 847/s ± 46/s and a minor state population of 1.63% ± 0.06% at 20.0 °C (Supplementary Fig. S7), suggesting that all these amide groups are involved in the same conformational exchange process, even though their location is non-contiguous in the tertiary structure (Fig. 5). With a root mean square deviation (RMSD) of 4.42 ppm, the ^15^N chemical shift changes deviate markedly from those expected for a random coil^35^ and are therefore inconsistent with a global or local unfolding process (Supplementary Fig. S8). Identification of the nature of this extensive conformational exchange process (for example, transient self-association or concerted/allosteric motions) has to await systematic quantification by relaxation dispersion spectroscopy for different nuclei at a series of different temperatures, which is beyond the scope of the present work.

**Figure 6:**
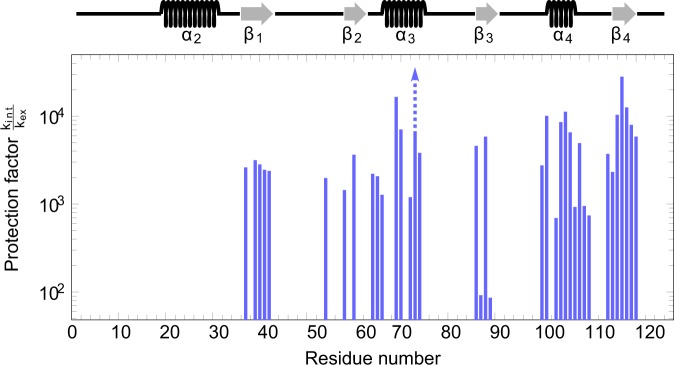
Logarithmic plot of the protection factors, PF = k_int_/k_ex_, calculated from the apparent backbone amide proton/deuteron (H/D) exchange rates, k_ex_, as measured in a series of [^1^H-^15^N] HSQC experiments at 20.0 °C (Supplementary Figs S9 and S10). Amide protons with protection factors below about 1 × 10^2^ exchange in less than half an hour and hence too rapidly to be observed with sufficient resonance intensity for quantification. By contrast, amide protons involved in hydrogen bonding in the regular secondary structure elements, which are indicated above the graph, are highly protected from solvent exchange (PF ≈ 5 × 10^2^ to 3 × 10^4^). The backbone amide proton of Ile73 exchanges so slowly (Supplementary Fig. S9) that only an upper bound for k_ex_ and hence a lower bound for PF (indicated by the arrow) could be determined.

Conformational exchange processes can also affect the backbone amide H/D exchange rates, k_ex_. Amide protons involved in stable hydrogen bonds are protected from exchange with the solvent and therefore show very large protection factors, PF = k_int_/k_ex_, which measure how many times slower the protons exchange with the solvent in NMR buffer than under denaturing (random coil) conditions. However, if such a hydrogen-bonded (“closed”) conformation C is in equilibrium with an alternate conformation O that is not hydrogen-bonded (“open”) then solvent exchange will readily occur via the fraction p_O_ of the protein that populates the alternate conformation. In the case of conformational exchange on the micro- to millisecond time-scale at pH 6.0 and 20.0 °C it can be shown^36^ that the resulting protection factors should be of the order of the inverse of the fractional population of the “open” state, PF ≈ 1/p_O_. As expected, the distribution of the highly protected backbone amide groups primarily reflects the hydrogen bonds stabilizing the regular secondary structure elements of LC3C (Fig. 6). By contrast, none of the amide groups in the flexible terminal regions is sufficiently protected from solvent exchange to allow experimental detection. Note that most of the amide protons in the loops identified above as “hotspots” of the conformational exchange on the millisecond time-scale also appear to exchange readily with the solvent (Fig. 6), indicating that the hydrogen bonding network of the regular secondary structure elements is stable and preserved by the conformational exchange processes; indeed, the high protection factors PF ≈ 2 × 10^3^ to 3 × 10^4^ found in all regular secondary structure elements except helix α_2_ are at variance with those expected for an “open” state with a fractional population p_O_ ≫ 0.1% large enough to give rise to significant exchange line broadening. In particular, this also rules out global unfolding of LC3C as the source of the observed exchange line broadening.

### Comparison with X-ray structures

**Figure 7:**
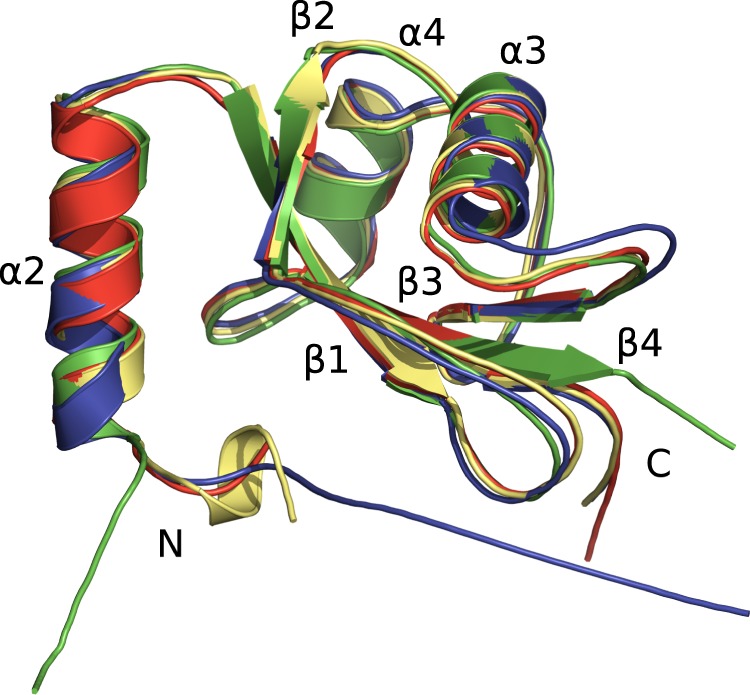
Comparison of a schematic representation of the secondary structure elements of the average solution structure of LC3C (green) with the crystal structures of LC3C in the free form (PDB 3WAM^14^, yellow; backbone RMSD 0.65 Å for residues Ser18 to Ala120) as well as in complex with NDP52 (PDB 3VVW^15^, red; 0.89 Å) and in complex with Atg13 (PDB 3WAP^14^, blue; 1.09 Å). The overlay and the calculation of backbone RMSDs were performed using PyMOL (The PyMOL Molecular Graphics System, Version 1.7.2.1, Schrödinger, LLC).

Overall, the NMR solution structure is in excellent agreement with the X-ray structures published for free LC3C(8–125)^14^ (PDB 3WAM), LC3C in complex with NDP52^15^ (PDB 3VVW), and LC3C(8–125) in complex with the LIR of Atg13^14^ (PDB 3WAP) (Fig. 7), with RMSDs of 0.65 Å, 0.89 Å, and 1.09 Å, respectively, for the backbone atoms of the structural core from Ser18 to Ala120. Minor differences are seen for the loops between strands β_1_ and β_2_ (Tyr44 to Thr56) and between helix α_3_ and strand β_3_ (Met77 to Phe85), which are involved in crystal contacts in the X-ray structures^14^ and undergo backbone motions on the pico- to nanosecond time-scale in solution (see above, Fig. 3). The largest differences, however, are observed for the NTR from the N-terminus to Lys17 (Fig. 7). In the crystal structure of LC3C in complex with NDP52^15^ (PDB 3VVW) residues Met1 to Pro12 could not be observed in the electron density map, consistent with our observation that this region exhibits no long-range order relative to the structural core of the protein (see above). The crystal structures of LC3C(8–125) in the free form and in complex with Atg13^14^ (PDB 3WAM and 3WAP, respectively) lack the additional six residues - including the polyproline motif - at the N-terminus of LC3C compared to LC3A and LC3B entirely (Fig. 1). In the absence of Atg13 no electron density could be found for residues before Arg11 but an additional helix α_1_ is formed from Pro12 to Lys17, which is also found in many other proteins of the Atg8 family^14^. Whether this difference in conformation is a consequence of the different crystallization conditions, the different crystal packing, the different resolution of the electron density maps, or the presence of the LIR of Atg13 remains unclear. In solution, no medium-range NOE pattern characteristic of such an additional helix α_1_ could be identified (Supplementary Fig. S5), and the low RCI order parameters S_RCI_^2^ are not indicative of any stable secondary structure in this region (Fig. 3D). However, some of the resonances in this region could not be identified in the NMR spectra^27^, most likely as a result of exchange line broadening due to conformational heterogeneity, so it is possible that this region transiently forms a metastable α-helical conformation. In addition to the N-terminal region, there are also conformational differences in the C-terminal region: Whereas the C-terminal residues Ser121 to Gly126 fold back towards the face of the central β-sheet in the crystal structures (Fig. 7), this region is poorly defined in solution (Fig. 2) by the sparse medium- and long-range NOEs (Supplementary Fig. S5), which reflects high mobility on the ps to ns time-scale (see above) and line broadening due to conformational exchange on the μs to ms time-scale (Figs 3A and 5). The mobility of the C-terminus in solution ensures that Gly126 (which is notably absent in the construct LC3C(8–125) used for two of the crystal structures^14^) is readily accessible for lipid conjugation by the ATG7/ATG3 system.

### Phosphorylation

In contrast to the GABARAP-like protein subfamily, the LC3 proteins share an amino-terminal, conserved PKA phosphorylation site^22^. In LC3C, the target residue is Ser18, whose hydroxyl group provides the N-capping hydrogen bond acceptor^37^ for helix α_2_ (see above). We have used a series of [^1^H-^15^N] HSQC spectra to follow chemical shift changes of the LC3C backbone amide groups during incubation with PKA *in vitro*. Subsequently, after >72 h, *in vitro* PKA-treated LC3C was proteolytically cleaved and analyzed by MALDI-LTQ-Orbitrap MS/MS. Glu-C hydrolysis of LC3C resulted in an amino-terminal fragment (GSMPPPQKIPSVRPFKQRKSLAIRQEE) for which a change in mass equivalent to the addition of a single phosphate group could be identified, and this fragment also gave rise to peaks with mass changes consistent with neutral losses of H_3_PO_4_ (mass change −97.97 Da) and HPO_3_ (mass change −79.96 Da) that are characteristic for phosphorylated peptides.

**Figure 8:**
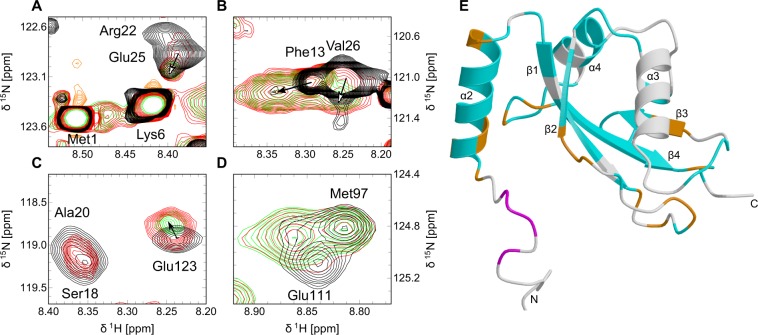
Overlay of sections of the [^1^H-^15^N] HSQC spectra (Supplementary Fig. S11) of [U-^13^C,^15^N] LC3C before (black) and 3.5 h (red) and 7.0 h (green) after incubation with PKA to monitor the progress of *in vitro* phosphorylation. (**A**) Intense resonance signals of Met1 and Lys6 did not shift, while the shift of the resonance of Glu25 could be traced (open arrow) and the resonance of Arg22 disappears. (**B**) The populations of the split signal of Phe13 in the unphosphorylated state (black) shifted towards the minor peak position (filled arrow), while the resonance of Val26 could be traced to a new peak position (open arrow). (**C**) The intensities of the resonances of Ser18 and Ala20 decreased within 3.5 h and could not be traced to newly emerging peaks in the vicinity, in contrast to Glu123 (filled arrow) and (**D**) Glu111. The resonance of Met97 was not influenced by PKA reaction progress. (**E**) Split backbone amide resonances (magenta), traceable backbone amide resonances (cyan), and disappearing backbone amide resonances (orange) after incubation with PKA color-coded onto a schematic representation of the secondary structure of the lowest-energy solution structure of LC3C (same view as in Fig. 5). The figure was drawn with MolScript 2.1.2^100^ and rendered with Raster3D 3.0^101^.

The signal-to-noise ratio of the [^1^H-^15^N] HSQC spectra gradually decreased to approximately a quarter of its original value over the incubation period of 63 h, although the solution remained clear and no new set of peaks with random coil chemical shifts that would indicate denaturation of the protein sample was observed to emerge. Initial chemical shift changes of several backbone amide resonances were already observed within the first 3.5 h after addition of PKA (Supplementary Fig. S11), but no further changes were detected after 10.5 h. Four qualitatively different patterns of chemical shift changes were found for different sets of residues: (i) Many of the backbone amide resonances show no significant chemical shift changes at all, including the intense resonances of the mobile amino-terminal residues from Met1 to Val10 (Fig. 8A), Met97 (Fig. 8D), and the carboxy-terminal residues Thr124 and Gly126, indicating that the conformation of these regions is unaffected by phosphorylation. (ii) By contrast, the amide groups of residues Arg11 to Gln15 exhibit two separate resonances with similar intensities in the presence of PKA, one at the position of the resonance in the unphosphorylated form plus an additional one with slightly different chemical shifts that appears to be present only with extremely low intensity close to the detection limit in the unphosphorylated form, as seen in Fig. 8B for Phe13. Accordingly, the effect of phosphorylation of Ser18 on this region appears to be an increase in the population of an alternate conformation, which exchanges with the conformation dominating in the unphosphorylated form on a time-scale of about 10 ms or slower, or not at all. It is possible that this alternate conformation is essentially the additional helix α_1_ that this region forms in the crystal structure of LC3C(8–125) (see above), although the relatively small chemical shift changes are also compatible with a subtler change in conformation. Note that two separate sets of NMR resonances resulting from slow conformational exchange are also observed for many residues located in or near helix α_1_ of GABARAP, which does not share the PKA phosphorylation site (Fig. 1), in the absence of PKA^9^. (iii) Another large set of backbone amide resonances, such as those of Glu25 (Fig. 8A), Val26 (Fig. 8B), Asp110, Glu111 (Fig. 8D), Gly113, and Glu123 (Fig. 8C) display a gradual but complete transition over the first 10.5 h of incubation with PKA from the chemical shifts of the unphosphorylated form to a newly emerging resonance with chemical shifts close enough to the original resonance to be readily traced. (iv) Finally, another set of backbone amide resonances including Ser18 (Fig. 8C), Ala20 (Fig. 8C) and Arg22 (Fig. 8A) in the immediate vicinity of the phosphorylation site, as well as Ala31 and Lys32 at the C-terminal end of helix α_2_, Tyr44, Arg46, Phe49 and Leu50 in the loop connecting strands β_1_ and β_2_, Lys55 to Lys57 in the LIR binding region, Tyr86, Leu87, Asp112, Phe114, and Ser121 disappear within 7.0 h after addition of PKA without any new resonance with similar chemical shifts emerging. We cannot rule out that amide resonances for these residues in the phosphorylated protein appear elsewhere in the spectrum because the quality of the NMR spectra of phosphorylated LC3C under these buffer conditions is insufficient for multidimensional assignment experiments. Alternatively, the disappearance of these resonances could be caused by unfavorable relaxation properties due to sample aggregation or chemical exchange on the μs to ms time-scale. The location of these resonances in the tertiary structure (orange in Fig. 8E) is intriguingly similar to the “hotspots” of conformational exchange identified by CPMG relaxation dispersion spectroscopy (Fig. 5), suggesting that the conformational effects of phosphorylation by PKA might be coupled to the conformational dynamics of unphosphorylated LC3C. Either way, the observed chemical shift changes indicate that phosphorylated LC3C is even more conformationally heterogeneous than unphosphorylated LC3C.

**Figure 9:**
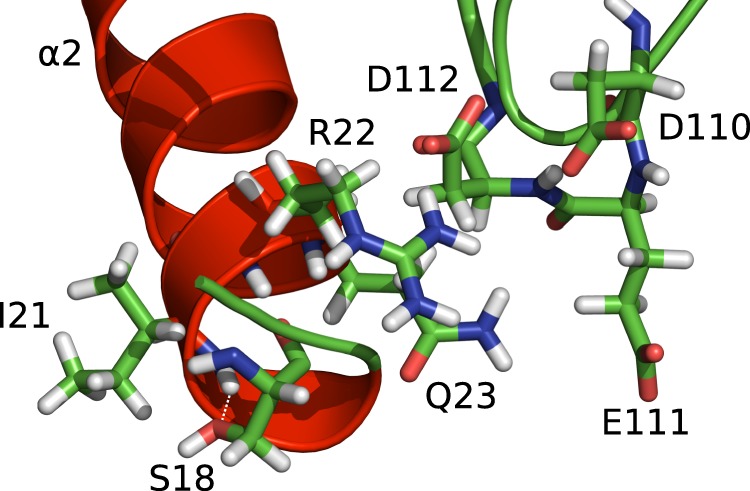
Electrostatic interactions of the acidic cluster formed by the side-chains of Asp110, Glu111, and Asp112 with helix α_2_ residues Arg22 and Gln23 in the crystal structure of LC3C(8–125) (PDB 3WAM^14^; hydrogen atoms were added with PyMOL). The backbone amide proton of Ile21 forms an N-capping motif with the side-chain hydroxyl oxygen of phosphorylation target Ser18 (dashed line). The figure was created with PyMOL (The PyMOL Molecular Graphics System, Version 1.7.2.1, Schrödinger, LLC).

As described above, the side-chain of Ser18 provides an N-capping hydrogen bond acceptor (Fig. 9), so phosphorylation of Ser18 could have an influence on the stability of this hydrogen bond and, in turn, on the stability of helix α_2_ in general, maybe even trigger a helix-to-coil transition^37^. Moreover, helix α_2_ is held in place by several favorable tertiary contacts, most notably electrostatic interactions of the side-chain of Arg22 with the negatively charged side-chains of Asp110, Glu111, and Asp112 (Fig. 9) in the loop between helix α_4_ and strand β_4_, a rigid loop that shows little sign of backbone mobility on the ps to ns time-scale nor of line broadening from conformational exchange on the μs to ms time-scale (Figs 2, 3, 5). The chemical shift changes of the backbone amide resonance from Asp110 to Gly113 upon addition of PKA (see above) suggest that the negative charge of the phosphate group modification modulates these interactions.

To test the plausibility of these hypotheses we performed comparative molecular dynamics (MD) simulations of LC3C in the unmodified (Ser18) and phosporylated (pSer18) form. In the context of a random-coil peptide, the pK_a_ of phosphoserine is very close to 6.0, the pH of the NMR buffer used here, and the exchange between the protonated (monoanionic) and deprotonated (dianionic) states is fast on the chemical shift time-scale^38^. Although both the pK_a_ and the exchange rate of the protonation equilibrium can be modulated by tertiary interactions we have to assume that both states are populated to a considerable degree in our NMR experiments^39^; in fact, exchange between these two protonation states might well be the cause underlying some of the line broadening or peak splitting in the NMR spectra of phosphorylated LC3C described above. Therefore, we calculated three MD trajectories of 100 ns each, one with the unmodified serine (Ser18), one with the protonated phosphoserine (pSer18^−^), and one with the deprotonated phosphoserine (pSer18^2−^). The hydrogen bond between the amide group of Ile21 and the side-chain of Ser18 is indeed formed a considerable fraction of the time along the MD trajectory (Supplementary Fig. S12) but virtually absent from the MD trajectories for pSer18^−^ (Supplementary Fig. S13) and pSer18^2−^ (Supplementary Fig. S14), which corroborates our hypothesis that this hydrogen bond is destabilized by phosphorylation by PKA. However, helix α_2_ remains stable for the entire duration of the MD trajectories regardless of the phosphorylation state of Ser18 (Supplementary Figs S12–S14) and no helix-to-coil transition is observed, at least not on the time-scale of 100 ns covered by the MD simulations. Instead of forming a hydrogen bond with the amide group of Ile21 the single negative charge of the monoanionic phosphate group of pSer18^−^ favors a salt bridge with the positively charged side-chain of Lys17 (Supplementary Fig. S13); intriguingly, the additional helix α_1_ is populated a significant fraction of the MD trajectory calculated with pSer18^−^ (Supplementary Fig. S13), in support of our suspicion that phosphorylation of Ser18 might stabilize a helical conformation in this region (see above). Of course, these MD simulations do not rule out the possibility of any additional conformational changes on a time-scale slower than about 100 ns that might be triggered by phosphorylation. For example, the disappearance of the amide resonances of Ala31 and Lys32 at the C-terminal end of helix α_2_ could readily be explained if this region acts as a hinge for repositioning of helix α_2_. In light of our experimental observation that the chemical shifts of several resonances in the LIR binding region of LC3C are strongly affected by phosphorylation (see above) a modulating effect of PKA on both, affinity as well as specificity of LC3C for its target LIRs appears highly likely.

## Conclusions

High-resolution structure determination by NMR spectroscopy confirms that the well-ordered core (residues 18 to 120) of the autophagy-related protein MAP1LC3C adopts virtually the same tertiary structure in solution as in X-ray crystallography, although extensive exchange line broadening reveals the existence of alternate, low-populated conformations. By contrast, both termini are highly mobile. The mobility of the C-terminus guarantees full access of the lipid conjugation machinery to the C-terminal residue for membrane anchoring of LC3C. The N-terminus forms a polyproline II helix (Pro2, Pro3, Pro4) tethered to the ubiquitin-like core via a flexible linker, an arrangement that has been described as a “sticky arm” in the literature^40^. Short proline-rich regions are a highly diverse protein-protein interaction motif found in a large number of proteins, often recognized by dedicated protein-protein interaction domains such as WW domains, SH3 domains, or profilins^40^. In this context, ligand specificity is usually conferred by the spacing of the proline residues as well as the biophysical properties of the side-chains flanking the prolines, and sometimes further modulated by phosphorylation. While the N-terminal sequence motif of LC3C, MPPPQK (Fig. 1), deviates from the stereotypical WW and SH3 domain ligand motifs as compiled by Kay *et al*.^40^, interfacing to such a protein-protein interaction domain cannot be ruled out as the functional role of the N-terminal PPII motif of LC3C. Intriguingly, though, proline-rich regions have also been identified to mediate interactions with microtubules, more specifically with the β-tubulin subunit^41^. Flexible proline-rich regions are involved in the interaction of the intrinsically disordered microtubule-associated protein Tau with microtubules, and phosphorylation has recently been shown to change the conformation and microtubule interaction mode of the second proline-rich region of Tau^42,43^. The full name MAP1LC3 (microtubule associated proteins 1 light chain 3) reflects the fact that this subfamily was originally identified as proteins associated with microtubules^44^, and the interplay between autophagy and microtubules is well-documented^45^, albeit still poorly understood. In the case of GABARAP the interaction with the negatively charged microtubular surface was mapped to the positively charged helix α_2_ with 7 basic residues^46^. Because this motif appears to be absent from LC3C, where only 3 of these 7 basic residues are conserved in helix α_2_ from Ser18 to Phe33 (Fig. 1), it is plausible to hypothesize that the divergence of the amino-terminal region across the human GABARAP/MAP1LC3 family (Fig. 1) provides for differential interaction with the cytoskeleton. The PKA phosphorylation site at Ser18 at the N-cap of helix α_2_ of LC3C is well-positioned to further modulate such an interaction. The chemical shift changes upon phosphorylation do not indicate a direct influence on the PPII motif itself but are consistent with repositioning of helix α_2_ and possibly also stabilization of a short additional helix α_1_ in the NTR. Phosphorylation of Ser18 also has a large effect on the chemical shifts in the LIR binding region of LC3C and can therefore be expected to modulate affinity and specificity of the interaction of LC3C with its target LIRs or CLIRs.

## Methods

### Cloning, expression and purification

LC3C was cloned, expressed and purified as an amino-terminal GST-LC3C fusion protein as described in detail previously^27^. Thrombin cleavage and final purification by cation exchange and size exclusion chromatography yielded highly pure samples of the cytosolic LC3C protein of 126 amino acids with a two-residue amino-terminal cloning artifact (Gly-1 and Ser0) (Supplementary Figs S1 and S2), which were concentrated to between 370 μM and 700 μM for NMR spectroscopy.

### NMR spectroscopy

NMR samples of 370 μM to 700 μM [U-^15^N] or [U-^13^C,^15^N] LC3C were prepared in 20 mM PIPES, 150 mM NaCl, 0.1 mM EDTA and supplemented with 2% (v/v) glycerol-d_8_ (Euriso-top, Gif-sur-Yvette, France) and 10% (v/v) ^2^D_2_O at pH 6.0 (NMR buffer). The effect of the protein concentration and of the presence or absence of 2% (v/v) glycerol-d_8_ on the position and qualitative line shape features of the NMR resonances is negligible (Supplementary Fig. S3). NMR experiments were recorded at 20.0 °C on Varian INOVA or Bruker AVANCE III spectrometers operating at ^1^H frequencies of 600 MHz and 800 MHz and equipped with cryogenically cooled triple or quadruple resonance probes with pulse-field gradient capabilities. Sequence-specific ^1^H, ^15^N, and ^13^C backbone and side-chain NMR resonance assignments and ^3^J_HNH__α_ scalar coupling constants were reported previously (BMRB 26603)^27^. ^1^H-^1^H distance information was derived from the following nuclear Overhauser effect spectroscopy (NOESY) experiments: 2D [^1^H-^1^H] NOESY^47^ (120 ms mixing time) in 99% ^2^D_2_O, 3D [^1^H-^15^N] NOESY-HSQC^48^ (120 ms mixing time) in 90% ^1^H_2_O/10% ^2^D_2_O, 3D [^1^H-^13^C] NOESY-HSQC^49^ (120 ms mixing time) in 99% ^2^D_2_O, 3D [^1^H-^15^N] [^1^H-^15^N] HSQC-NOESY-HSQC^50,51^ (150 ms mixing time) in 90% ^1^H_2_O/10% ^2^D_2_O, and 3D [^1^H-^13^C] [^1^H-^15^N] HSQC-NOESY-HSQC^52^ (150 ms mixing time) in 90% ^1^H_2_O/10% ^2^D_2_O. The ^1^H_2_O resonance was suppressed by excitation sculpting^53^ in the 2D [^1^H-^1^H] homonuclear experiments and by gradient coherence selection in the heteronuclear experiments, quadrature detection in the indirect dimensions was achieved by States-TPPI^54^ or the echo/antiecho method^55,56^. All NMR spectra were processed using NMRPipe and NMRDraw^57^ and analysed with NMRViewJ^58^ and CcpNmr Analysis^59^.

To measure amide proton/deuteron (H/D) exchange a sample of 570 μM [U-^15^N] LC3C in NMR buffer was freeze-dried and a series of seven consecutive [^1^H-^15^N] HSQC experiments was recorded 0.3, 1.6, 2.8, 5.4, 7.9, 12.9, and 17.9 h after reconstitution of the lyophilized sample in ^2^D_2_O. Signal intensities in the resulting [^1^H-^15^N] HSQC spectra were quantified by three-way decomposition using MUNIN^60,61^ and fitted by mono-exponential decay functions using the Levenberg-Marquardt algorithm as implemented in MATLAB R2015b (The MathWorks, Inc., Natick, Mass., USA) to extract amide H/D exchange rates for semi-quantitative analysis^62^. Experimental H/D exchange rates, k_ex_, were converted into protection factors PF = k_int_/k_ex_, where k_int_ are the intrinsic (unprotected) H/D exchange rates for these experimental conditions as predicted from the amino acid sequence^63^ using the SPHERE server (http://landing.foxchase.org/research/labs/roder/sphere).

Protein backbone amide group dynamics on the pico- to nanosecond time-scale was probed by ^15^N spin relaxation experiments^34,64^ recorded on a sample of 510 μM [U-^15^N] LC3C in NMR buffer at 800 MHz and 20.0 °C. ^15^N longitudinal relaxation rates, R_1,_ were obtained from ^15^N inversion recovery experiments^64,65^ with 11 different inversion recovery times (3 of them collected in duplicate) between 80 ms and 1200 ms and a recycle delay of 2.5 s. ^15^N rotating frame relaxation rates, R_1ρ_, were obtained from ^15^N spin lock experiments^66^ with 10 different spin lock times (3 of them collected in duplicate) between 10 ms and 100 ms with a spin lock field strength of 2.02 kHz and a recycle delay of 3.0 s. Amide resonance intensities were quantified by three-way decomposition and fit by mono-exponential decay functions using MUNIN^60,61^ to extract the ^15^N relaxation rates R_1_ and R_1ρ_. ^15^N transverse relaxation rates, R_2_, were calculated from R_1_ and R_1ρ_^67^. {^1^H}^15^N heteronuclear NOE values were calculated as the amide resonance intensity ratios in a pair of interleaved spectra recorded with and without proton saturation by applying a train of 120° pulses at a field strength of 11.6 kHz for the final 6.0 s of the recycle delay of 15.0 s^64^. Uncertainties of the {^1^H}^15^N NOE values were estimated from the spectral noise background. The overall rotational diffusion tensor and “model-free” parameters^30^ describing internal motion of the protein backbone such as the generalized order parameters for sub-nanosecond internal motion, S^2^, were determined by fitting the experimental ^15^N R_1_ and R_2_ rates and {^1^H}^15^N NOE values using Tensor 2.0^68^ with the default parameters based on the lowest-energy structure of LC3C. Amide groups with {^1^H}^15^N NOE values below 0.65 and/or with R_2_/R_1_ ratios deviating by more than 10% from the mean value were considered to possess significantly increased internal mobility and excluded from the calculation of the rotational diffusion tensor^29^. The presence of millisecond time-scale exchange processes was probed by ^15^N single-quantum Carr-Purcell-Meiboom-Gill (CPMG) relaxation dispersion experiments^69,70^ recorded on this sample at 600 MHz as well as 800 MHz and 20.0 °C. In each ^15^N CPMG experiment 17 (14) different CPMG frequencies ν_CPMG_ = 1/(2δ), where δ is the time between consecutive refocusing pulses, ranging from 41.7 Hz (50.0 Hz) to 2000.0 Hz (1000.0 Hz) were sampled during a constant-time relaxation interval of T_CPMG_ = 48 ms (40 ms) at 600 MHz (800 MHz). Amide resonance intensities I(ν_CPMG_) were quantified by three-way decomposition using MUNIN^60,61^ and converted into effective transverse relaxation rates, R_2,eff_(ν_CPMG_) = −ln(I(ν_CPMG_)/I_0_)/T_CPMG_, where I_0_ is the corresponding resonance intensity in a reference spectrum recorded without the constant-time relaxation interval. Error estimates ΔR_2,eff_ for R_2,eff_ were obtained from duplicate measurements at 3 different ν_CPMG_ values as described previously^71^, assuming a minimum relative error ΔR_2,eff_/R_2,eff_ of 2.0% to account for offset effects and other systematic experimental imperfections. Global exchange parameters (exchange rates, equilibrium populations) and residue specific values (^15^N chemical shift differences, Δϖ_N_, intrinsic relaxation rates, R_2,0_) were extracted by a non-linear least-squares fitting procedure whereby experimental dispersion profiles, R_2,eff_(ν_CPMG_) = R_2,0_ + R_ex_(ν_CPMG_), were fit by those calculated from the evolution of magnetization during the CPMG interval by solving the Bloch-McConnell equations numerically for a two-site exchange model as described previously^71,72^. Errors of the fitted parameters were calculated from the covariance matrix^73^, an approach that has been shown to provide reasonable error estimates^74^.

### Structural restraints and structure calculation

Based on the almost complete assignment of the ^1^H, ^15^N, and ^13^C resonances of LC3C published previously^27^, the NOE cross peaks in the [^1^H-^15^N] and [^1^H-^13^C] NOESY-HSQC spectra were quantified with CcpNmr Analysis 2.4.1^59^ and automatically assigned and converted into NOE distance restraints using ARIA 2.3^75,76^ in an iterative procedure. All resulting NOE assignments were inspected manually. 94 ^3^J_HNH__α_ coupling constants were obtained from a quantitative HNHA experiment with a coherence transfer time of 12.3 ms and relaxation correction factor of 1.1^77,78^ as described previously^27^. 32 of these ^3^J_HNH__α_ in the well-ordered core of the protein were sufficiently different from the value of 7.0 Hz that is indicative of rotameric averaging^79^ and had sufficiently small experimental uncertainties to be converted into Φ backbone torsion angle restraints using the Karplus relation^77^ as implemented in CcpNmr Analysis with a tolerance of ±30°. Additional Φ and Ψ backbone dihedral angle restraints were derived from the secondary chemical shifts (^1^HN, ^1^Hα, ^15^N, ^13^CO, ^13^Cα, ^13^Cβ) by TALOS-N^80,81^. Amide hydrogen bond donors were identified as slowly exchanging protons in the H/D exchange experiment and the corresponding hydrogen bond acceptors were found by proximity in later stages of the structure calculation process and then incorporated as additional distance restraints. For each of the 51 hydrogen bonds the distance between the amide proton and the acceptor was restrained to between 1.5 Å and 2.3 Å and the distance between the amide nitrogen and the acceptor to between 2.5 Å and 3.5 Å. These experimental restraints served as input for the calculation of 100 structures using restrained molecular dynamics simulations with ARIA-optimized CNS 1.21^82^ using the CNS protocol parameters listed in Supplementary Table S1. The 10 structures showing the lowest energy values were further refined in an explicit water shell using the CSDX/OPLS hybrid force field as implemented in ARIA/CNS^83^ and selected for further characterization. Structural models were visualized by PyMOL (The PyMOL Molecular Graphics System, Version 1.7.2.1, Schrödinger, LLC) and analyzed using ARIA/CNS, the NIH version 1.2.1^84^ of X-PLOR 3.851^85^, PROCHECK-NMR^86^, MolProbity^87^, and CING^88^.

### *In-vitro* phosphorylation

LC3C samples were phosphorylated *in vitro* using the catalytic subunit of murine PKA (NEB, Frankfurt a. M., Germany). A 650 μM sample of [U-^13^C, ^15^N]-labeled LC3C in NMR buffer was supplemented with 4 mM MgCl_2_ and subsequently 1 mM ATP (PKA buffer). This change in buffer conditions was monitored by [^1^H-^15^N] HSQC spectra to rule out any major spectral differences that might indicate a substantial modulation of the structure or dynamics of LC3C. Finally, 1 μl PKA (circa 2500 U) was added and the reaction was monitored by a series of 17 consecutive [^1^H-^15^N] HSQC experiments at 800 MHz and 20.0 °C over a period of 63 h. Additionally, a [^1^H-^13^C] ct-HSQC experiment was recorded at 800 MHz and 20.0 °C after six days of incubation. Furthermore, the phosphorylation reaction was analyzed by proteolytic hydrolysis with trypsin and Glu-C of the [U-^13^C,^15^N]-labeled reaction product, followed by MALDI-LTQ-Orbitrap MS/MS analysis.

### Molecular dynamics simulations

The lowest-energy solution structure after water refinement was used as a starting structure for molecular dynamics (MD) simulations. Three independent phosphorylation states of LC3C were simulated: unmodified serine 18 (Ser18), serine 18 mutated to a protonated phosphoserine (pSer18^−^), and serine 18 mutated to a deprotonated phosphoserine (pSer18^2−^). The Amber force field ff99SB-ILDN^89^ was used in conjunction with the TIP3P water model^90^. Parameters for bonded and van der Waals interactions of the phosphorylated serine residue were taken from the GAFF force field^91^ generated with acpype.py^92^ and the partial charges from Homeyer *et al*.^39^. All MD simulations were performed using GROMACS 5.1^93^. The protein was centered in a dodecahedral box with a minimum solute-to-wall distance of 1 nm. Sodium chloride ions were added to neutralize the systems and mimic a salt concentration of approximately 100 mM. The systems were first energy-minimized, equilibrated in the NPT ensemble (i. e., with a constant number of molecules, pressure, and temperature) for 1 ns and then simulated for 100 ns each in the NPT ensemble at 298 K (Nosé-Hoover thermostat^94^) and 1.0 bar (Parrinello-Rahman barostat^95^). Van der Waals and short-range electrostatic interactions were cut off at 12 Å and long-range electrostatics were treated with the particle mesh Ewald method^96^. Bond lengths and bond angles of hydrogen atoms were constrained to their equilibrium values with the LINCS algorithm^97^. The equations of motion were integrated with a velocity Verlet integrator and a time step of 2 fs. Atom positions were saved every 10 ps.

## Supplementary information

Supplementary Info

## Data Availability

The atomic coordinates and experimental restraints have been deposited with the Protein Data Bank (access code: 2NCN; DOI: http://dx.doi.org/10.2210/pdb2ncn/pdb).
