# Supplementary material for: Solution structure of the autophagy-related protein LC3C reveals a polyproline II motif on a mobile tether with phosphorylation site
Source: Sci Rep. 2019 Oct 2;9:14167. doi: 10.1038/s41598-019-48155-8 (PMC6775092; doi:10.1038/s41598-019-48155-8)
Supplement: Supplementary file 1 — Supplementary Info [file 41598_2019_48155_MOESM1_ESM.pdf]

Solution structure of the autophagy-related protein LC3C reveals a polyproline II motif on a mobile tether with phosphorylation site

Carsten Krichel[1,2], Christina Möckel[1,2], Oliver Schillinger[1,3], Pitter F. Huesgen[4], Heinrich Sticht[5], Birgit Strodel[1,3], Oliver H. Weiergräber[1], Dieter Willbold[1,2] & Philipp Neudecker[1,2]

[1]ICS-6 (Strukturbiochemie), Forschungszentrum Jülich, 52425 Jülich, Germany

[2]Institut für Physikalische Biologie and BMFZ, Heinrich-Heine-Universität Düsseldorf, 40225 Düsseldorf, Germany

[3]Institut für Theoretische Chemie und Computerchemie, Heinrich-Heine-Universität Düsseldorf, 40225 Düsseldorf, Germany

[4]ZEA-3 (Analytik), Forschungszentrum Jülich, 52425 Jülich, Germany

[5]Institut für Biochemie, Friedrich-Alexander-Universität Erlangen-Nürnberg, 91054 Erlangen, Germany

Correspondence should be addressed to D.W. (email: d.willbold@fz-juelich.de) or P.N. (email: p.neudecker@fz-juelich.de)

Supplementary Information

Supplementary Tables

Table S1. Overview of molecular dynamics parameters used for structure calculation by CNS 1.21

Parameter	Value
TAD high temperature	10000 K
TAD time-step factor	9.0
Cartesian high temperature	2000 K
Cartesian 1st iteration	0
Time-step	3 fs
Cool1 final temperature	1000 K
Cool2 final temperature	50 K
High-temp steps	10000
Refine steps	4000
Cool1 steps	20000
Cool2 steps	20000

Supplementary Figures

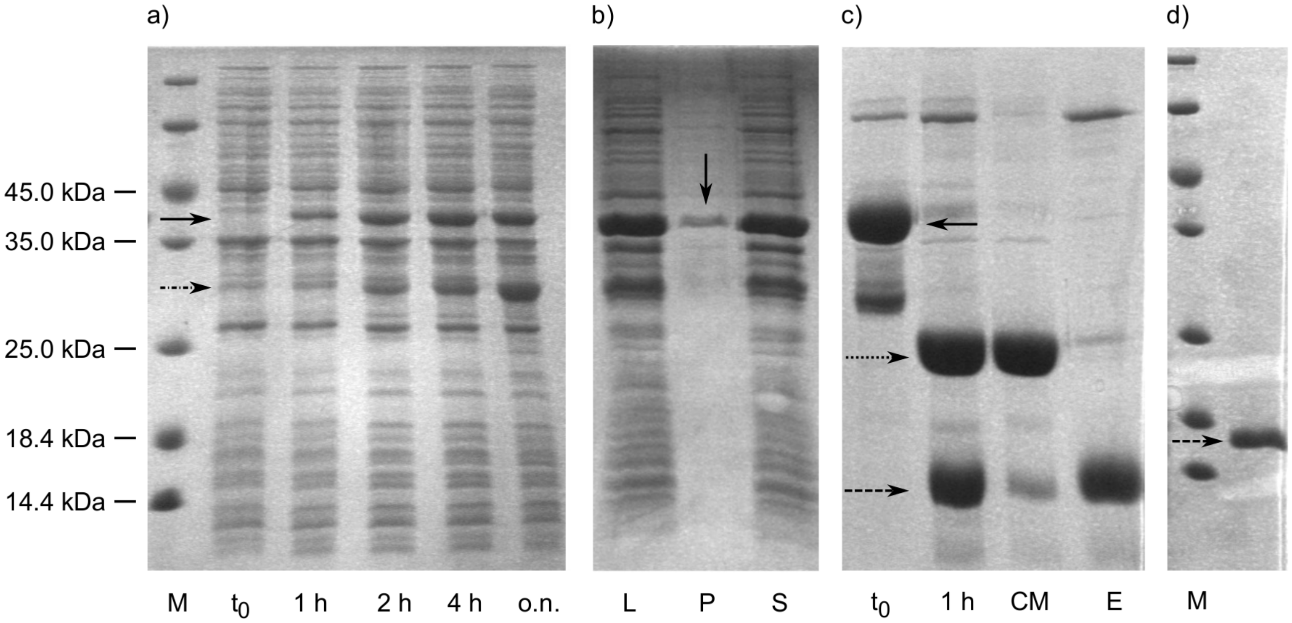

Figure S1. Purification of LC3C documented by SDS-PAGE. (**a**) Expression of GST-LC3C fusion protein (41.4 kDa, marked by arrow) at 25°C. M - protein marker, t_0 - before induction, 1 h/2 h/4 h - time points after induction, and o.n. - overnight expression. (**b**) Cell lysis, L - whole cell lysate, P - unsoluble pellet after centrifugation of lysate, S - supernatant. (**c**) On-column hydrolysis of GST-LC3C fusion protein by bovine thrombin at room temperature. t_0 - column material with bound GST-LC3C fusion protein (marked by arrow) before addition of thrombin, 1 h - resuspended column material one hour after addition of thrombin (GST marked by dotted arrow, LC3C marked by dashed arrow), CM - remaining column material after elution, E - elution fraction containing cleaved LC3C. (**d**) Purified LC3C after cation exchange and size exclusion chromatography steps (marked by dashed arrow).

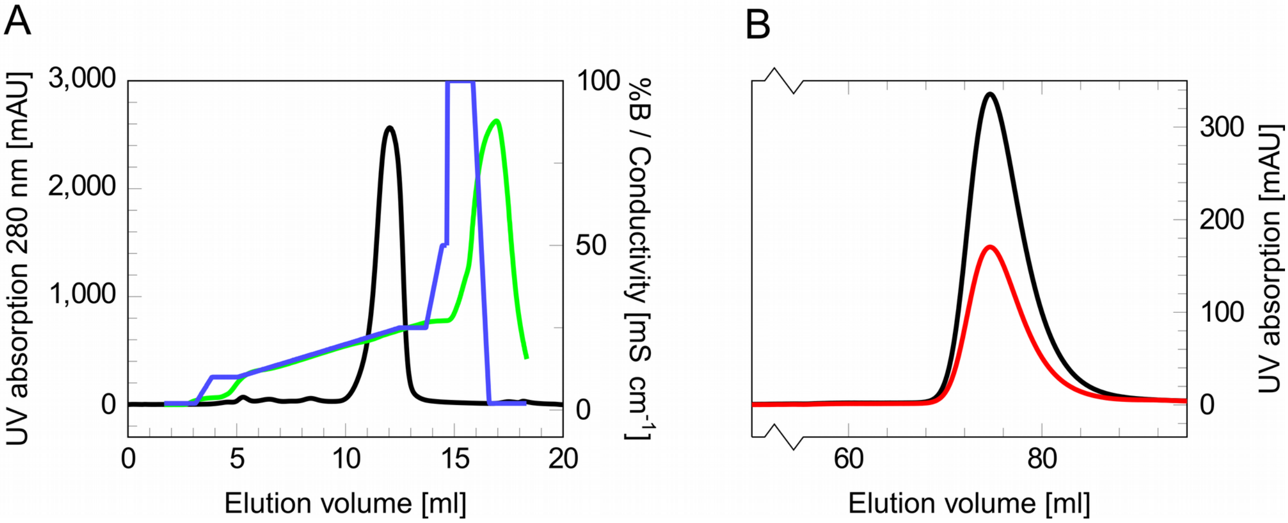

Figure S2. Overview of LC3C chromatographic purification. (**A**) Cation exchange chromatogram of LC3C (Resource S column in a buffer of 20 mM PIPES at pH 6.0 at a flow rate of 1.5 ml/min at room temperature). UV absorption recorded at 280 nm (black), high-salt gradient from 0.0 M (0% elution buffer B) to 1.0 M NaCl (100% elution buffer B consisting of 1.0 M NaCl, 20 mM PIPES, pH 6.0) (blue), and conductivity of eluent (green). (**B**) Size exclusion chromatogram of LC3C (Superdex 75 16/60 at a flow rate of 1.5 ml/min at 8°C). UV absorption recorded at 280 nm and 320 nm (black and red, respectively).

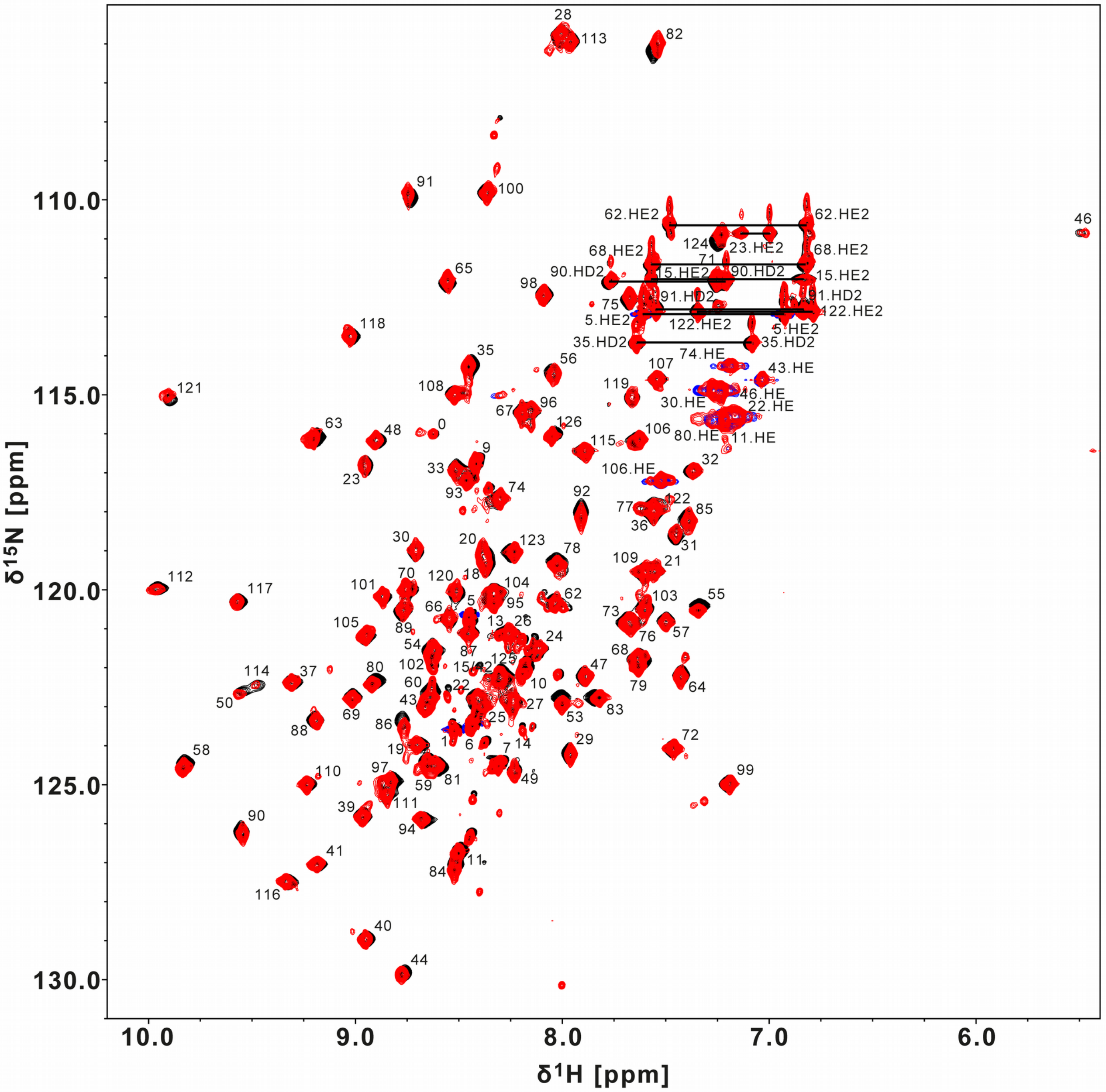

Figure S3. Overlay of the [^{1}H-^{15}N] HSQC spectra of 560 μM [U-^{15}N] LC3C in 20 mM PIPES, 150 mM NaCl, 0.1 mM EDTA, 2% (v/v) glycerol-d$_8$, 10% (v/v) ^{2}D$_2$O, pH 6.0 recorded at 20.0°C and 600 MHz (positive contours black, negative contours blue) and of 65 μM [U-^{15}N] LC3C in 20 mM PIPES, 150 mM NaCl, 0.1 mM EDTA, 10% (v/v) ^{2}D$_2$O, pH 6.0 recorded at 20.0°C and 800 MHz (positive and negative contours red). Backbone resonance assignments[27] are indicated by residue numbers. Side-chain resonances of Asn and Gln residues are connected by horizontal lines, amine side-chain resonances of Arg are aliased in the ^{15}N dimension and appear as negative contours. Backbone resonances of Arg16 and Lys17 could not be identified due to severe exchange line broadening[27]. Peak positions and qualitative line shape features such as dynamic narrowing and exchange broadening are virtually identical in the presence and absence of 2% (v/v) glycerol-d$_8$ in the buffer and at concentrations differing by almost an order of magnitude.

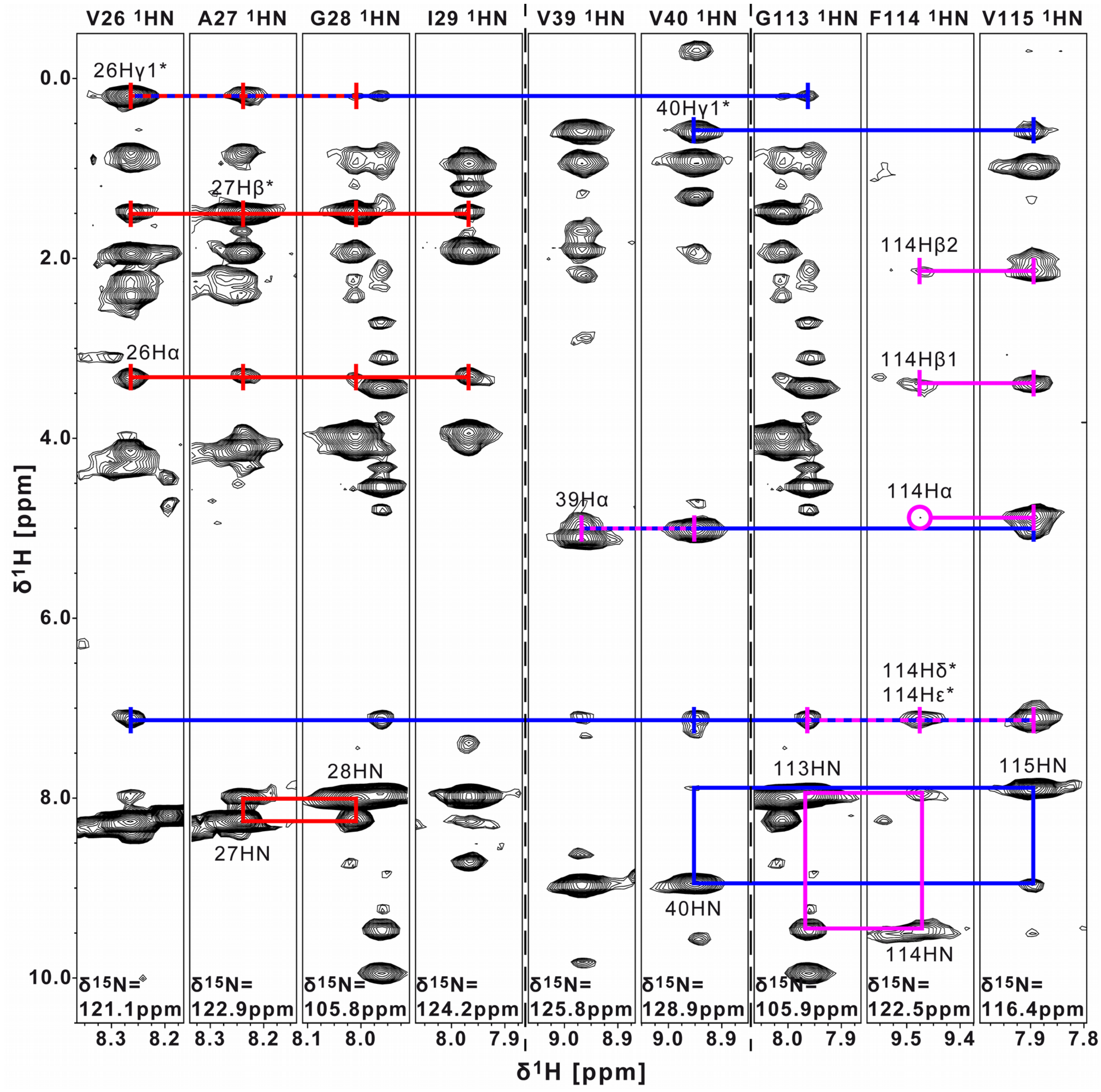

Figure S4. NOE cross peaks of the backbone amide protons of Val26 to Ile29 (located in helix α_2), Val39 to Val40 (in strand β_1), and Gly113 to Val115 (at the N-terminus of strand β_4) in the 3D [^{1}H-^{15}N] NOESY-HSQC recorded at 20.0°C and 600 MHz. The strips are taken at the backbone amide ^{15}N chemical shift, δ^{15}N (as indicated in the bottom of each strip), and are 0.20 ppm wide in the directly detected amide proton dimension, δ^1H (abscissa). A selection of sequential and medium-range NOEs in α-helices and β-strands is highlighted by red and magenta lines, respectively, and the NOE partner is labeled in its intraresidual strip. Selected long-range NOEs highlighted by blue lines reflect the parallel strand pairing between β_1 and β_4 as well as the tertiary contact between helix α_2 and the loop at the N-terminus of strand β_4 (see Fig. 2). Note that the NOE cross peaks of Phe114 are significantly attenuated by exchange line broadening (compare Fig. S3).

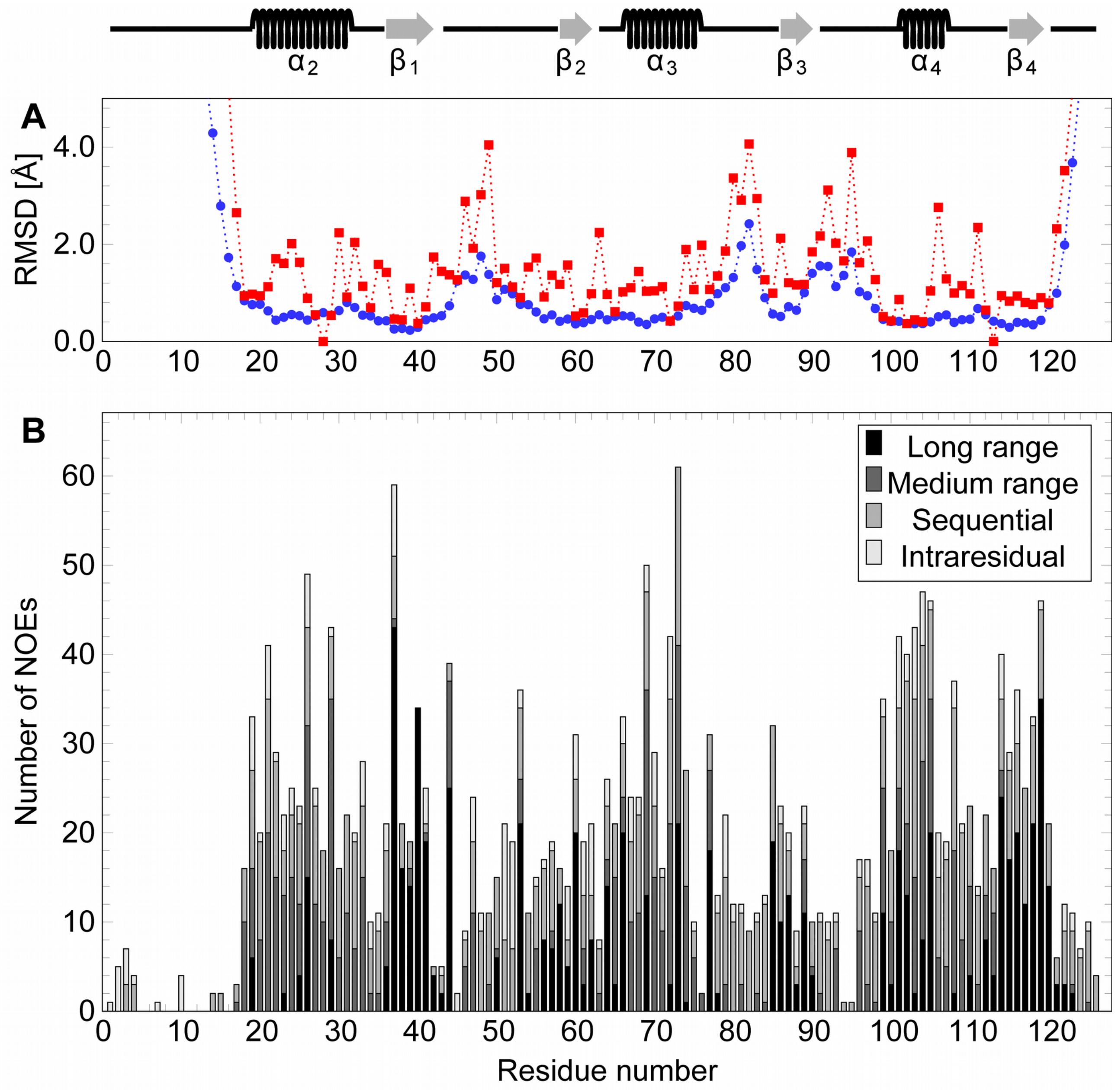

Figure S5. Atomic RMSDs from the average structure (**A**) and distribution of NOEs (**B**). Backbone RMSDs are indicated by blue circles, side-chain heavy atom RMSDs by red squares. The regular secondary structure elements are indicated above the graph.

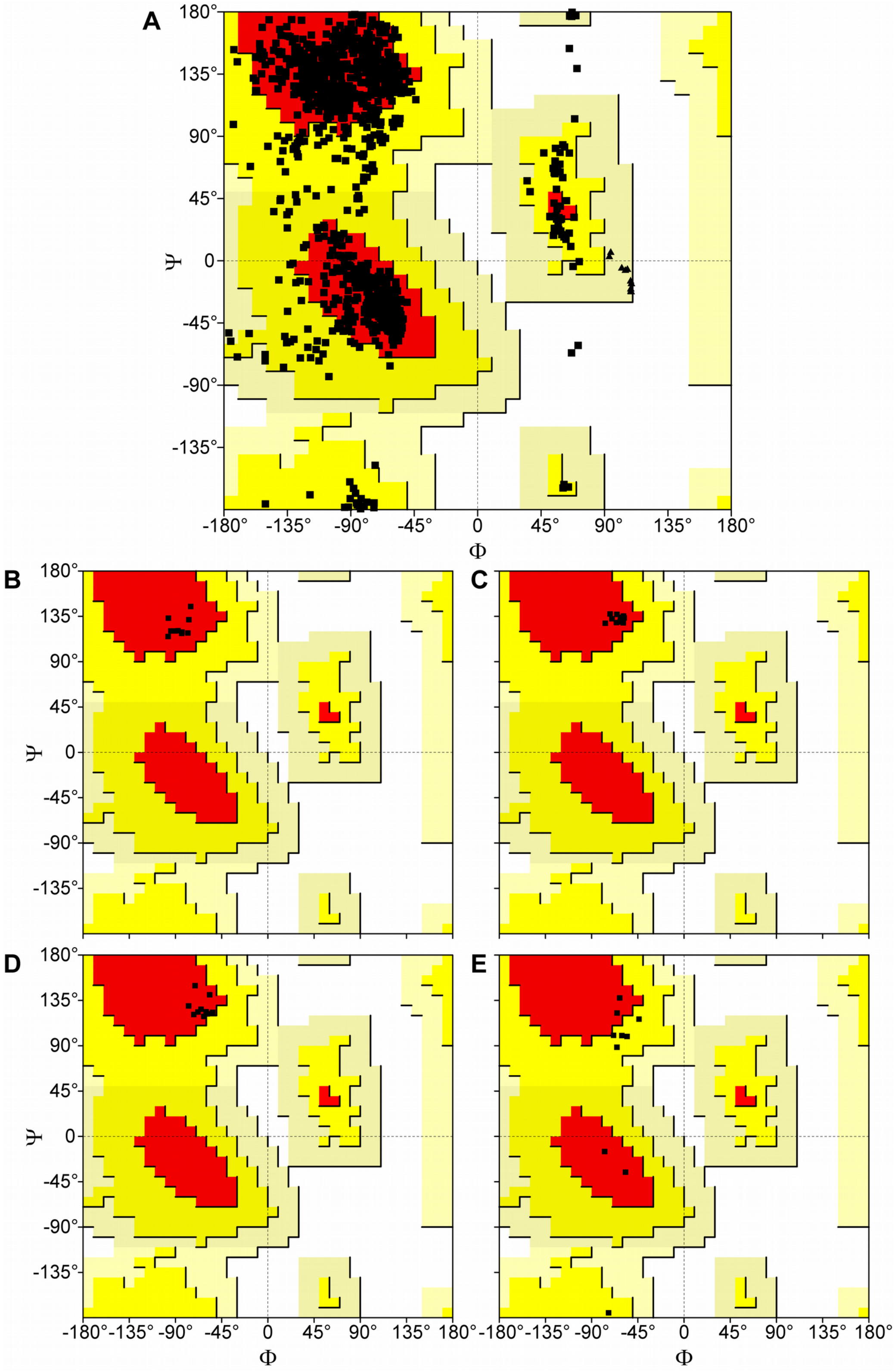

Figure S6. Ramachandran plots of the 10 accepted structures for (**A**) all residues, (**B**) Met1, (**C**) Pro2, (**D**) Pro3, and (**E**) Pro4. Glycine and non-glycine residues are represented by black triangles and squares, respectively. The polyproline II (PPII) helix exhibits characteristic backbone torsion angles of $\Phi \approx -75°$ and $\Psi \approx +120°$; note that the Ψ angle of Pro4 is not part of the PPII motif itself but describes the mobile orientation of the PPII motif with respect to Gln5 (see Fig. 4). The figure was created using PROCHECK-NMR[86].

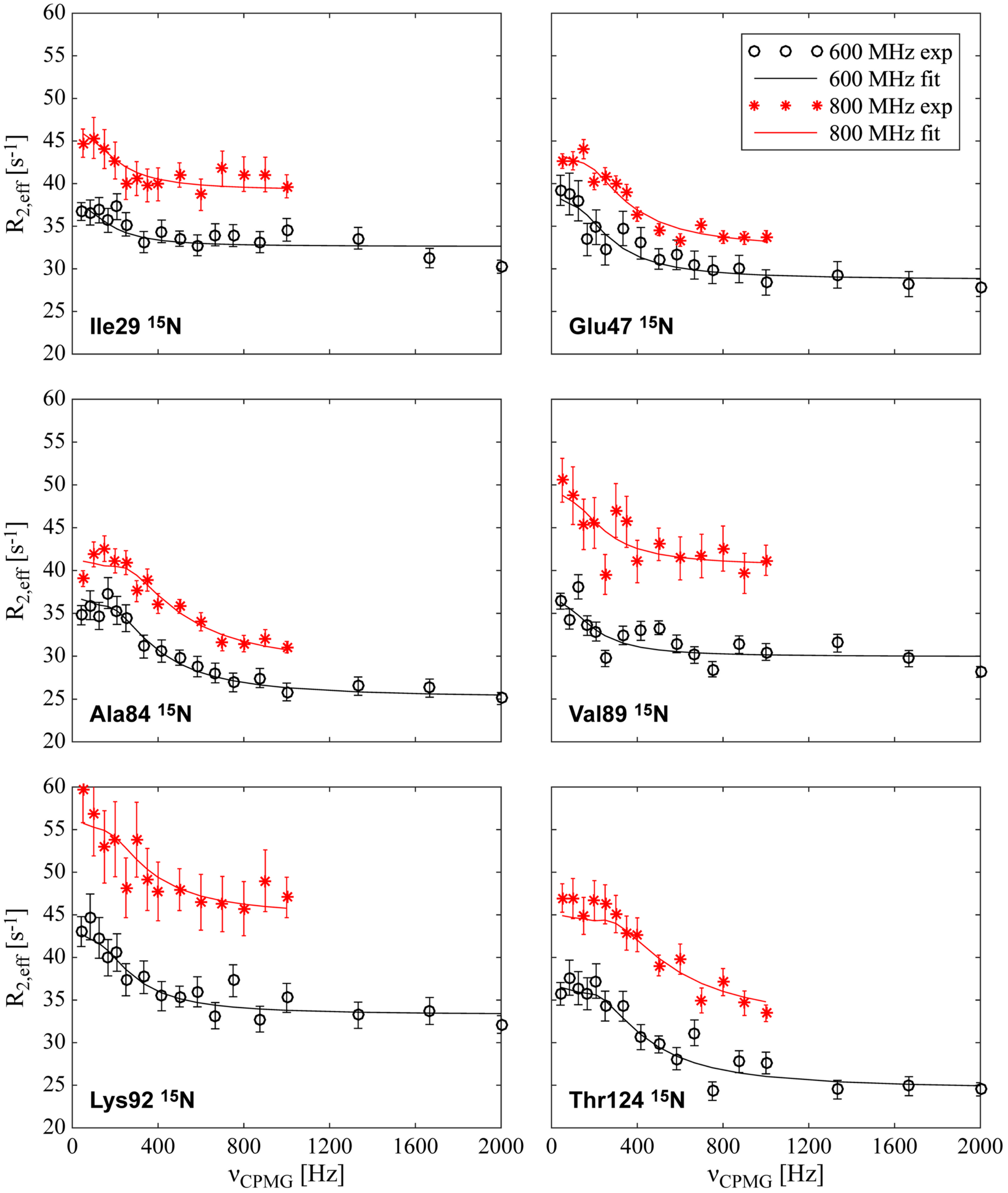

Figure S7. ^{15}N single-quantum relaxation rates $R_{2,eff}$ as a function of CPMG frequency ν_{CPMG} for Ile29 (helix α_2), Glu47 (loop between strands β_1 and β_2), Ala84 (loop between helix α_3 and strand β_3), Val89 (strand β_3), Lys92 (loop between strand β_3 and helix α_4), and Thr124 (C-terminal tail) at 800 MHz (red asterisks) and 600 MHz (black circles) and a temperature of 20.0°C. Solid lines are the relaxation dispersion profiles predicted using parameters generated from fitting the two-site exchange model described in the text to the ^{15}N relaxation dispersion data for all residues together.

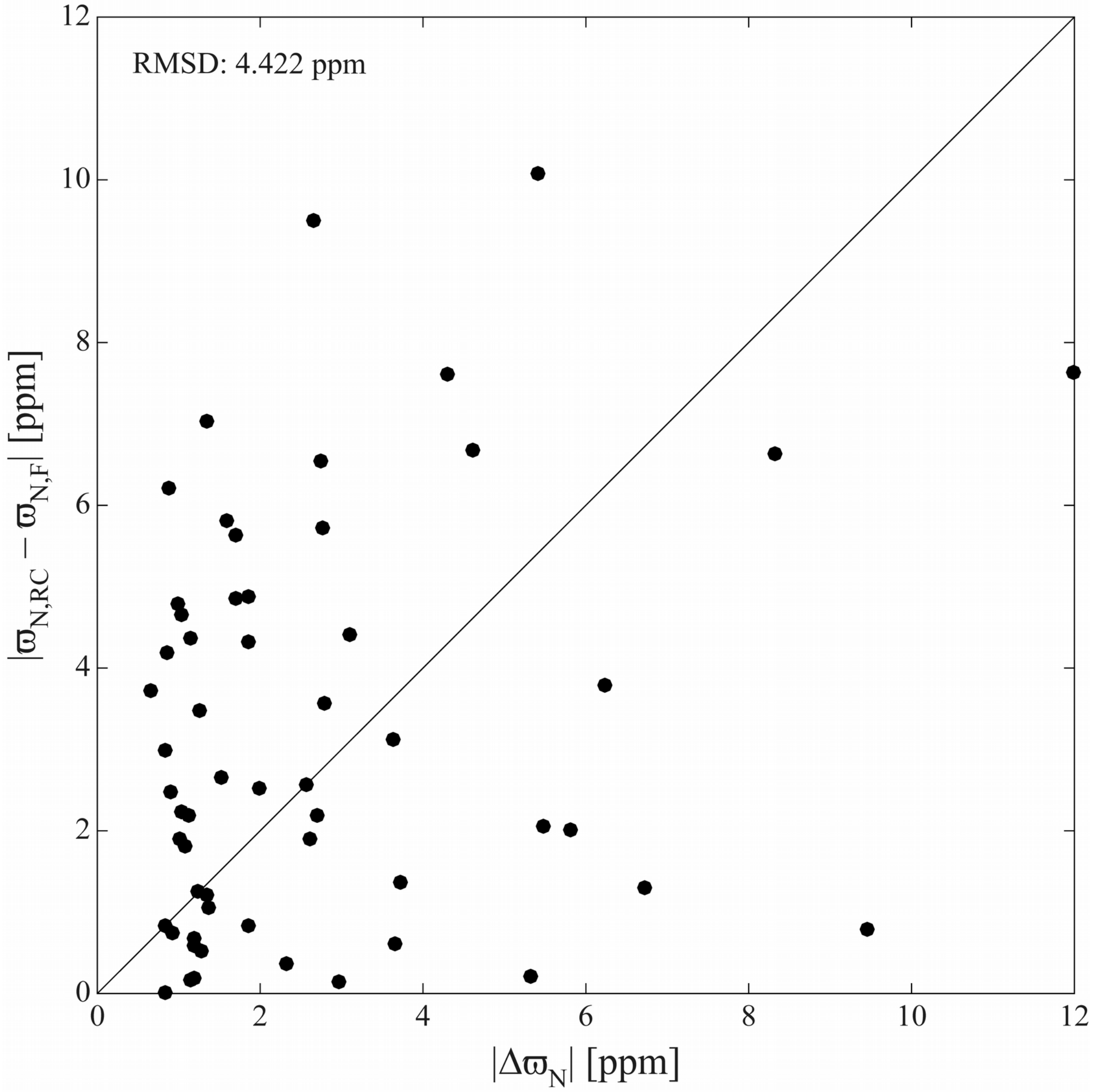

Figure S8. Comparison of the absolute value of the ^{15}N chemical shift changes, $|\Delta\varpi_N|$, extracted by fitting the CPMG relaxation dispersion data at 20.0°C using a two-site model of exchange, with the absolute value of the differences between random coil[35], $\varpi_{N,RC}$, and natively folded[27], $\varpi_{N,F}$, ^{15}N chemical shift values of LC3C. This plots establishes that the minor state at 20.0°C is not a random coil ensemble.

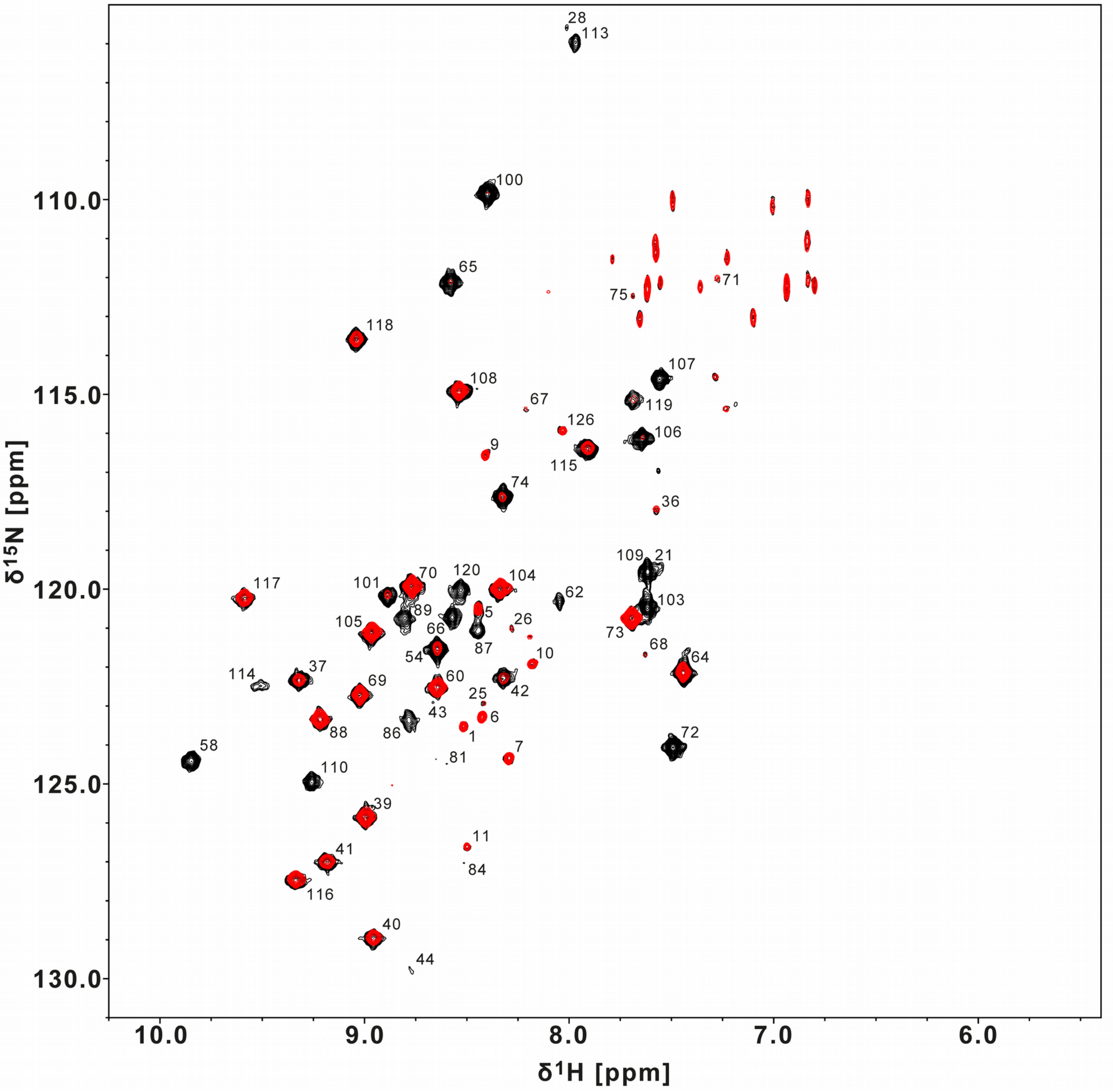

Figure S9. Overlay of the [^{1}H-^{15}N] HSQC spectra of 570 μM [U-^{15}N] LC3C in 20 mM PIPES, 150 mM NaCl, 0.1 mM EDTA, 2% (v/v) glycerol-d_8, pH 6.0 recorded at 20.0°C and 600 MHz 0.3 h (black) and 17.9 h (red) after reconstitution of the lyophilized sample in ^2D_2O. Backbone resonance assignments[27] are indicated by residue numbers. Backbone amide protons not protected by hydrogen bonds typically exchange with the deuterated solvent completely within minutes, including those from the flexible N-terminal region, whose relaxation properties are so highly favorable that the tiny residual equilibrium protonation level is observed nevertheless. By contrast, some amide protons such as that of Ile73 are so highly protected that they had barely exchanged with the deuterated solvent at all after 17.9 h.

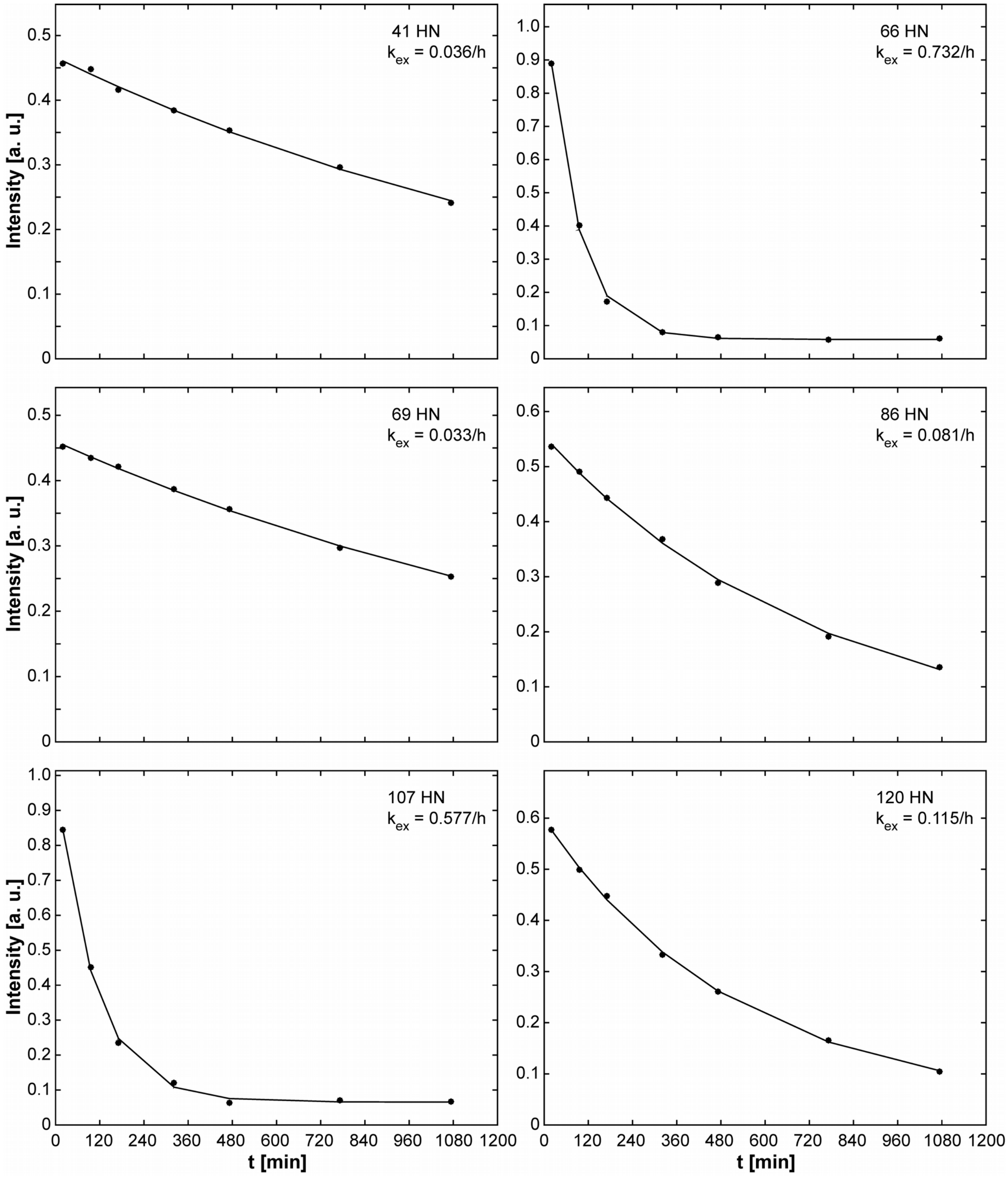

Figure S10. Determination of backbone amide H/D exchange rates, k_{ex}, by fitting mono-exponential decays (solid lines) to the amide resonance intensities (filled circles) in the [^{1}H-^{15}N] HSQC spectra recorded at time t after reconstitution of the lyophilized sample in ^2D_2O (Fig. S9) for Val41 (strand β_1), Met66 & Phe69 (helix α_3), Tyr86 (strand β_3), Asp107 (helix α_4), and Ala120 (strand β_4). These examples illustrate amide protons protected by H-bonds in different secondary structure elements and span over one order of magnitude in exchange kinetics. Non-linear least-squares fitting was performed using the Levenberg-Marquardt algorithm as implemented in MATLAB R2015b (The MathWorks, Inc.).

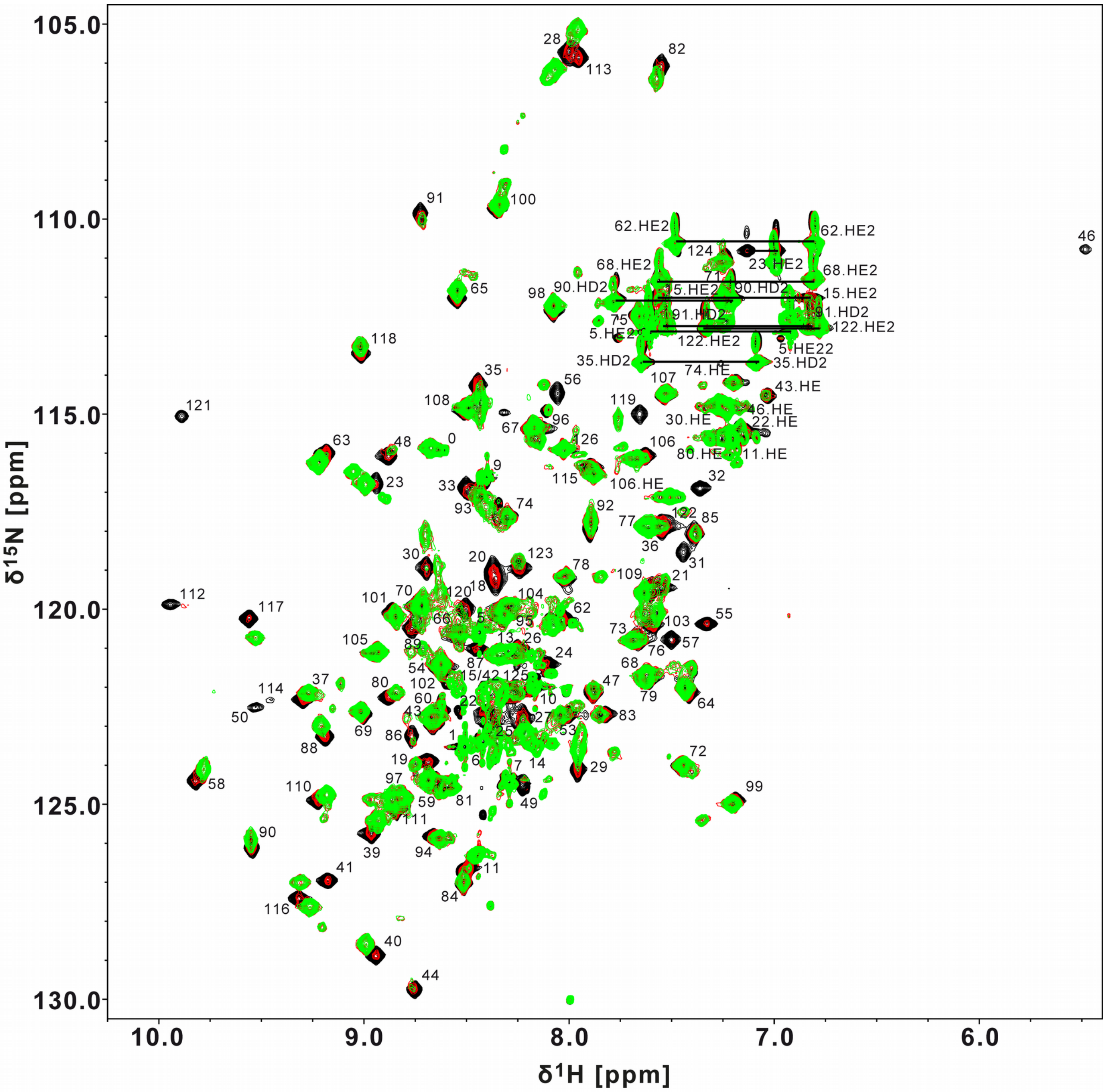

Figure S11. Overlay of the [^{1}H-^{15}N] HSQC spectra of 650 μM [U-^{13}C,^{15}N] LC3C in 20 mM PIPES, 150 mM NaCl, 0.1 mM EDTA, 2% (v/v) glycerol-d_8, 10% (v/v) $^{2}D_2O$, pH 6.0 with 4 mM $MgCl_2$ and 1 mM ATP recorded at 20.0°C and 800 MHz before (black) and 3.5 h (red) and 7.0 h (green) after addition of 1 μl (approx. 2500 U) PKA. Backbone resonance assignments[27] of unmodified LC3C are indicated by residue numbers. Side-chain resonances of Asn and Gln residues are connected by horizontal lines, amine side-chain resonances of Arg are aliased in the ^{15}N dimension.

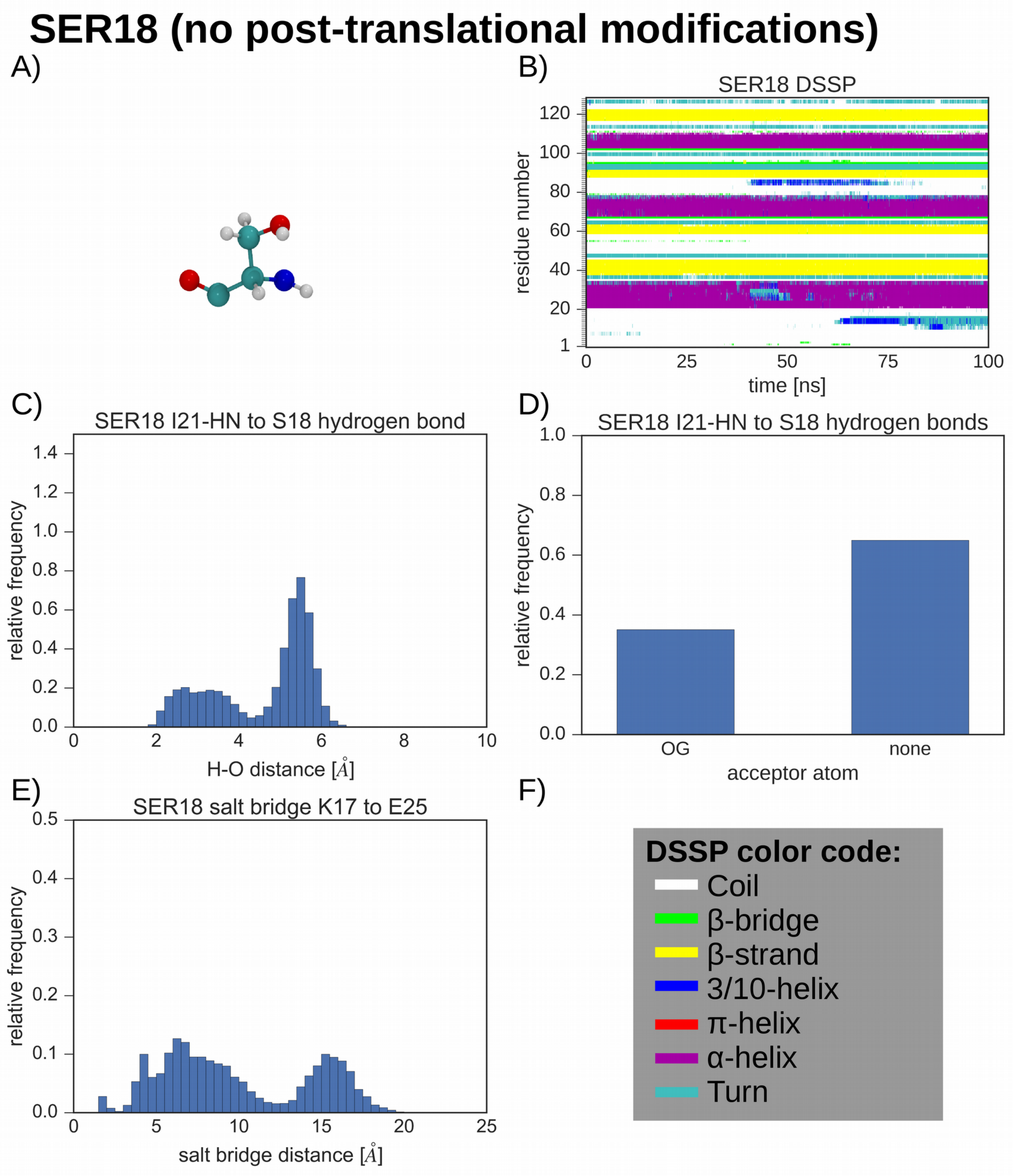

Figure S12. Analysis of the MD trajectory of unmodified LC3C. (**A**) Unmodified serine residue in ball-and-stick representation (C: cyan, N: blue, O: red, H: white). (**B**) Secondary structure elements along the MD trajectory as identified with DSSP[99] (see color code at the bottom). (**C**) Histogram of the distance between the backbone amide proton of Ile21 and the hydroxyl oxygen of Ser18. (**D**) Histogram of the hydrogen bond acceptor for the amide proton of Ile21 (OG: Ser18 Oγ). (**E**) Histogram of the salt bridge distance between Lys17 and Glu25. All histograms have been normalized to unit area.

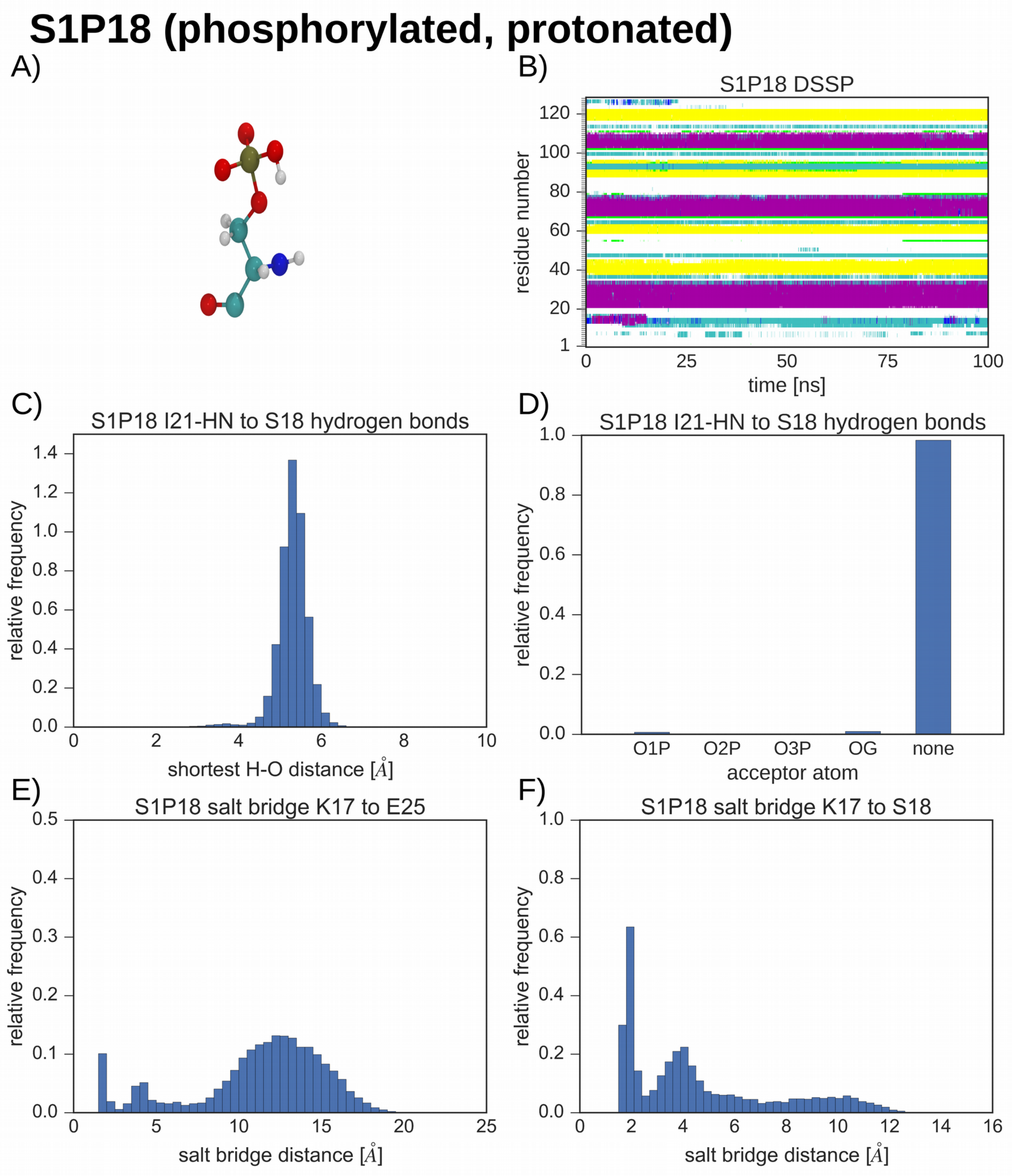

Figure S13. Analysis of the MD trajectory of phosphorylated LC3C in the monoanionic state. (**A**) Protonated phosphoserine residue in ball-and-stick representation. (**B**) Secondary structure elements along the MD trajectory as identified with DSSP. (**C**) Histogram of the shortest distance between the backbone amide proton of Ile21 and one of the four phosphate oxygens of pSer18^{-}. (**D**) Histogram of the hydrogen bond acceptor for the amide proton of Ile21 (OG: pSer18^{-} Oγ, O1P/O2P/O3P: pSer18^{-} terminal phosphate oxygens). (**E**) Histogram of the salt bridge distance between Lys17 and Glu25. (**F**) Histogram of the salt bridge distance between Lys17 and pSer18^{-}. See legend to Fig. S12 for details.

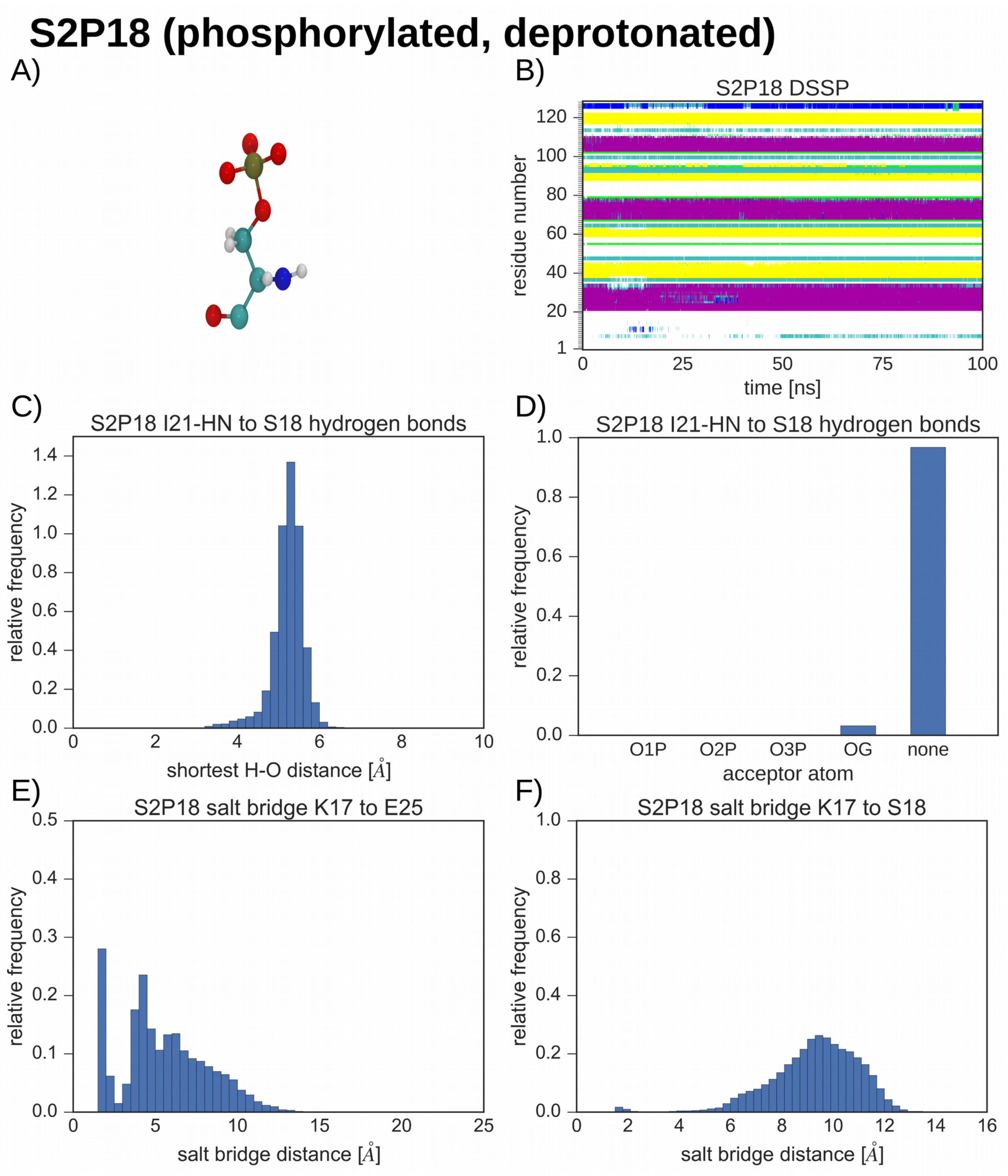

Figure S14. Analysis of the MD trajectory of phosphorylated LC3C in the dianionic state. (**A**) Deprotonated phosphoserine residue in ball-and-stick representation. (**B**) Secondary structure elements along the MD trajectory as identified with DSSP. (**C**) Histogram of the shortest distance between the backbone amide proton of Ile21 and one of the four phosphate oxygens of pSer18^{2-}. (**D**) Histogram of the hydrogen bond acceptor for the amide proton of Ile21 (OG: pSer18^{2-} Oγ, O1P/O2P/O3P: pSer18^{2-} terminal phosphate oxygens). (**E**) Histogram of the salt bridge distance between Lys17 and Glu25. (**F**) Histogram of the salt bridge distance between Lys17 and pSer18^{2-}. See legend to Fig. S12 for details.
